# Selection of Priority VOC Emission Sources in Seoul by Comparing Source Contributions and Health Risk-Based Contributions

**DOI:** 10.3390/toxics14090799

**Published:** 2026-09-09

**Authors:** Ji-Yun Jung, Shin-Young Park, Dong-Seop Shin, Jong-Cheol Yoon, Hak-Myeong Lim, Kwang-Rae Kim, Seok-Ryul Oh, Yong-Suk Choi, Hyun-Seok Cho, Cheol-Min Lee

**Affiliations:** 1 Department of Chemical and Environmental Engineering, Seokyeong University, Seoul 02713, Republic of Korea; jju1049@skuniv.ac.kr (J.-Y.J.); tlsdud060900@skuniv.ac.kr (S.-Y.P.); sds920703@gmail.com (D.-S.S.);; 2 Seoul Metropolitan Government Research Institute of Public Health and Environment, Seoul 13818, Republic of Korea; yoonkjc@seoul.go.kr (J.-C.Y.); chayke1@seoul.go.kr (H.-M.L.); kimkr@seoul.go.kr (K.-R.K.); ifnwu@seoul.go.kr (S.-R.O.); hozer87@seoul.go.kr (Y.-S.C.)

**Keywords:** volatile organic compounds, health risk assessment, source-oriented risk assessment, source contribution, risk-based source prioritization

## Abstract

This study conducted age-specific and source-oriented health risk assessments of volatile organic compounds (VOCs) at four measurement sites in Seoul (Gangseo (GS), Gwangjin (GJ), Bukhansan (BHS), and Jongno (JN)). By comparing source-specific concentration contributions with health risk-based contributions, priority sources and compounds were identified. The age-specific health risk assessment showed that, under the central tendency exposure (CTE) scenario, both the total excess cancer risk (TECR) and hazard index (HI) at all measurement sites remained below the recommended guideline values. However, under the reasonable maximum exposure (RME) scenario, TECR exceeded 1 × 10^−6^ for some adult and elderly groups, indicating the potential for adverse health effects. Compound-specific analysis identified Benzene and Ethylbenzene as the major contributors to TECR, whereas Ethylbenzene, Benzene, and Toluene were the primary contributors to HI. The source-oriented health risk assessment revealed that concentration-based and health risk-based source contributions exhibited different patterns, with manufacturing-related sources ranking among the highest contributors to health risks at all measurement sites. At the GS site, the highest-ranking sources in terms of concentration and health risk contributions were *Metal processing product manufacturing* and *Other product manufacturing*, respectively. At the GJ site, *Fuel-related emissions* ranked highest in concentration contribution, whereas *Metal processing products*, *chemicals and chemical products manufacturing* ranked highest in health risk contribution. At the BHS and JN sites, *Background hydrocarbons* showed the greatest concentration contribution, while *Chemicals and chemical products manufacturing*, *Vehicle exhaust: Acetylene-rich*, and *Textile product manufacturing* were the major contributors. This study demonstrates that VOC management in Seoul should prioritize health risk-based rather than concentration-based approaches.

## 1. Introduction

Volatile organic compounds (VOCs) are major precursors that play important roles in atmospheric photochemical reactions and are recognized as representative hazardous air pollutants that exert complex impacts on both human health and the atmospheric environment. VOCs are known to affect the nervous, respiratory, and cardiovascular systems, and several VOC species have been classified as carcinogenic substances by the International Agency for Research on Cancer (IARC). In addition, VOCs act as major precursors of Ozone (O_3_) and Secondary Organic Aerosols (SOAs), thereby contributing to the deterioration of air quality [1,2,3,4]. In particular, megacities such as Seoul, characterized by high population density and the coexistence of diverse emission sources, are likely to experience elevated health risks associated with VOC exposure. Furthermore, under the VOC-limited photochemical regime observed in Seoul, VOC reduction has been identified as a critical component of air quality management strategies [4,5].

To effectively manage atmospheric VOCs, extensive studies have been conducted on source identification and quantitative source contribution assessment based on receptor models [6]. Among various receptor models, the Positive Matrix Factorization (PMF) model has been most widely applied because it can identify major emission sources and estimate their contributions based on the statistical characteristics of measured concentration data. Accordingly, PMF has been widely utilized as a fundamental tool for establishing air quality management policies [7,8,9].

However, conventional source contribution-based approaches mainly focus on concentration contributions and therefore have limitations in directly reflecting human health impacts. In other words, even emission sources with relatively low concentration contributions may exert greater health impacts if they contain highly toxic compounds. Previous studies by Yang et al. [7], Zheng et al. [8], Xiong et al. [10], and Wang et al. [2] reported cases in which sources with relatively low total concentration contributions were identified as major contributors to carcinogenic risk, demonstrating discrepancies between concentration-based contributions and health risk-based contributions. Recently, increasing attention has been given to integrated approaches combining source contribution analysis with health risk assessment in order to address these limitations [10,11,12]. Nevertheless, in Korea, studies directly comparing source contribution and health risk contribution and systematically deriving management priorities based on these integrated analyses remain limited.

Accordingly, as part of an effort to overcome the limitations of concentration-oriented management approaches and to establish health impact-based VOC management strategies reflecting domestic emission characteristics, this study conducted health risk assessments based on adult and age-specific exposure scenarios using a Korean-specific VOC source classification framework previously established through the integration of PMF and PRTR data. Furthermore, by comparing source contribution estimates with health risk-based contributions, this study aimed to identify priority emission sources requiring management intervention.

## 2. Materials and Methods

### 2.1. Measurement Sites

**Figure 1:**
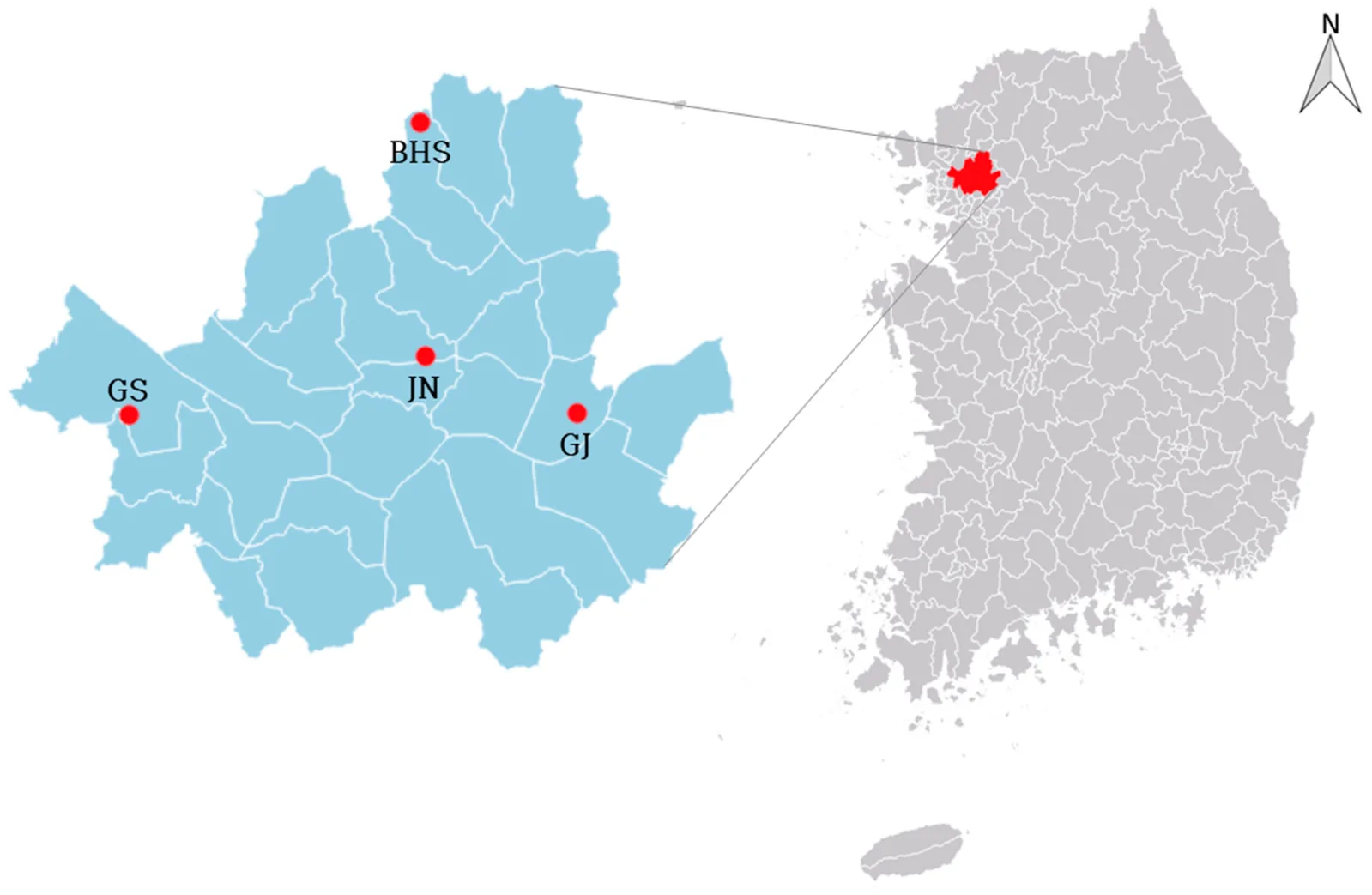
Measurement sites in this study (reproduced from Jung et al. [13]).

Hourly VOC observations from January 2018 to March 2024 were obtained from four PAMS sites operated by the Seoul Metropolitan Government Research Institute of Public Health and Environment: Gangseo (GS), Gwangjin (GJ), Bukhansan (BHS), and Jongno (JN). These sites were selected to represent contrasting urban emission environments across Seoul. GS reflects combined industrial and traffic influences in western Seoul, whereas JN represents a densely populated central urban area strongly affected by road traffic. GJ is characterized by mixed residential and commercial land use, while BHS, located in the northern mountainous area, was treated as a background site with relatively limited local emissions and greater potential influence from regional transport. This spatial contrast was used to evaluate whether source-specific health risk patterns differed according to local emission characteristics. The spatial distribution of the four measurement sties is illustrated in Figure 1.

### 2.2. Measurement and Analytical Methods

VOC monitoring and instrumental analysis were conducted according to the procedures used for the Seoul PAMS network, with detailed analytical conditions and QA/QC information provided in the Supplementary Materials. For the present health risk assessment, the original dataset containing 56 monitored VOC species was screened prior to analysis to obtain a consistently quantifiable dataset over the study period. Species were retained when at least 50% of their observations were above the method detection limit (MDL), with detection frequency calculated as the number of measurements exceeding the MDL relative to the total number of measurements [14]. Application of this screening criterion resulted in a final set of 16 VOCs. These consisted of eight alkanes (Ethane, Propane, Isobutane, n-Butane, Isopentane, n-Pentane, Hexane, and n-Decane), two alkanes (Ethylene and Propylene), one alkyne (Acetylene), and five aromatic compounds (Benzene, Toluene, Ethylbenzene, m/p-Xylene, and o-Xylene). Detection frequencies for all 56 monitored species and their inclusion or exclusion from subsequent analyses are reported in Table S3.

To evaluate the potential selection bias associated with the 50% detection frequency criterion, an additional sensitivity analysis was conducted for VOC species excluded from the primary analysis. For excluded VOCs with applicable inhalation toxicity reference values, concentrations below the site-specific MDLs were conservatively replaced with the corresponding MDL values, while measured concentrations at or above the MDLs were retained as observed. Health risks were subsequently recalculated using the same exposure assessment and risk characterization procedures as those applied in the primary analysis, and the resulting TECR and HI values were compared with the baseline estimates. Detailed procedures and results of the sensitivity analysis are provided in Section S4 of the Supplementary Materials.

### 2.3. Inhalation Health Risk Assessment of VOCs

Age-dependent differences in physiological and exposure-related characteristics can result in different inhalation doses even when individuals are exposed to the same ambient VOC concentrations. To account for this variability, inhalation health risks were evaluated separately for six age groups using group-specific exposure parameters. Both carcinogenic and non-carcinogenic risks were estimated under central tendency exposure (CTE) and reasonable maximum exposure (RME) scenarios [15].

#### 2.3.1. Hazard Identification

Hazard identification was performed for the 16 VOCs included in the analysis using inhalation toxicity information available in the US EPA Regional Screening Levels (RSLs) database. The toxicity values compiled in the RSLs are derived from multiple authoritative sources, including the Integrated Risk Information System (IRIS), Provisional Peer Reviewed Toxicity Values (PPRTVs), Agency for Toxic Substances and Disease Registry (ATSDR), California Environmental Protection Agency (Cal EPA), and Health Effects Assessment Summary Table (HEAST) [16]. Based on the toxicity information available as of April 2026, inhalation reference values applicable to the present assessment were available for eight compounds: Propylene, n-Pentane, Hexane, Benzene, Toluene, Ethylbenzene, m/p-Xylene, and o-Xylene.

#### 2.3.2. Dose–Response Assessment

Dose–response assessment was conducted using the Reference Concentration (RfC) and Inhalation Unit Risk (IUR) values available for the eight VOCs identified during hazard identification. The toxicity values used in this study are presented in Table 1. For subsequent dose-based risk calculations, RfC and IUR were converted to the corresponding Reference Dose (RfD) and Cancer Slope Factor (CSF) using Equations (1) and (2), respectively.

(1) $$RfD\left(mg/kg/day\right)=\frac{RfC(mg/m^{3})\times IRs}{BWs}$$

(2) $$CSF\left[{\left(mg/kg/day\right)}^{-1}\right]=\frac{IUR[(\mu g/m^{3}{)}^{-1}]\times BWs}{IRs}\times 1000\mu g/mg$$

In Equations (1) and (2), IRs and BWs denote the standard inhalation rate (20 m^3^/day) and standard body weight (70 kg), respectively, based on the values specified by the US EPA.

**Table 1:** Toxicity information of VOCs.

VOCs	IUR ^a^ ((μg/m^3^)^−1^)	RfC ^b^ (mg/m^3^)	Reference
Propylene	-	3.0 × 10^0^	Cal EPA
n-Pentane	-	1.0 × 10^0^	PPRTV
Hexane	-	7.0 × 10^−1^	IRIS
Benzene	2.2 × 10^−6^	3.0 × 10^−2^	IRIS
Toluene	-	5.0 × 10^0^	IRIS
Ethylbenzene	2.5 × 10^−6^	1.0 × 10^0^	Cal EPA, IRIS
m/p-Xylene	-	1.0 × 10^−1^	IRIS
o-Xylene	-	1.0 × 10^−1^	IRIS

^a^ IUR: Inhalation Unit Risk. ^b^ RfC: Reference Concentration.

#### 2.3.3. Exposure Assessments

Two exposure scenarios were established to reflect variability in ambient VOC exposure: CTE and RME. VOC concentrations corresponding to the 50th and 95th percentiles were used to represent the CTE and RME scenarios, respectively.

Age-specific exposure was evaluated by dividing the study population into six life-stage groups: infants (0–2 years), young children (3–6 years), elementary school children (7–12 years), adolescents (13–18 years), adults (19–64 years), and the elderly (65–84 years). The assessment was restricted to the inhalation pathway. Age-specific exposure time (ET) was assigned according to the daily time spent outdoors for each age group [17,18]. Exposure frequency (EF) was set at 300 days/year for the CTE scenario and 330 days/year for the RME scenario, following the exposure assumptions adopted from the NAS framework [15]. Exposure duration (ED) was defined by the number of years represented by each age interval, and the corresponding Average Time (AT) was expressed in hours based on the assigned ED. A lifetime (LT) of 84 years was adopted by rounding the 2024 Korean life expectancy of 83.7 years reported by the Korean Statistical Information Service [19]. For carcinogenic risk assessment, the estimated risk represents the lifetime average risk associated with exposure occurring during each defined age interval, rather than the probability of developing cancer within the remaining lifetime of an individual currently belonging to that age group. Therefore, differences in TECR among age groups are influenced by age-specific exposure parameters, particularly ED, IR, and BW, while the same LT averaging period (84 years) was applied to all age groups. The exposure factors used in this assessment are summarized in Table 2.

Exposure doses were quantified as the lifetime average daily dose (LADD) for carcinogenic effects and the average daily dose (ADD) for non-carcinogenic effects using the compound-specific concentrations and age-specific exposure factors (Equations (3) and (4)). Inhalation risk was evaluated using toxicity values derived specifically for the inhalation pathway. Consistent with the US EPA inhalation risk assessment framework, no compound-specific pulmonary absorption fractions were additionally applied because inhalation toxicity values such as RfC and IUR incorporate route-specific dosimetric considerations in their derivation.

(3) $$LADD\left(mg/kg/day\right)=\frac{C\times IR\times ET\times EF\times ED}{BW\times \mathrm{LT}}$$

(4) $$ADD\left(mg/kg/day\right)=\frac{C\times IR\times ET\times EF\times ED}{BW\times AT}$$

In Equations (3) and (4), C denotes the VOC concentration (mg/m^3^), while IR, ET, EF, ED, and BW represent the inhalation rate (m^3^/day), exposure time (h/day), exposure frequency (day/yr), exposure duration (yr), and body weight (kg), respectively. LT denotes lifetime (h), and AT represents the averaging time (h).

#### 2.3.4. Risk Characterization

Compound-specific health risks were characterized separately for carcinogenic and non-carcinogenic effects. Excess cancer risk (ECR) was estimated from the product of LADD and the corresponding cancer slope factor (CSF), whereas the hazard quotient (HQ) was obtained as the ratio of ADD to the reference dose (RfD), as shown in Equations (5) and (6), respectively.

(5) ECR=LADD×CSF

(6) $$HQ=\frac{ADD}{RfD}$$

Cumulative health risks from simultaneous exposure to multiple VOCs were assessed following the US EPA approach for chemical mixtures [20]. For carcinogenic effects, compound-specific ECR values were aggregated to derive the total excess cancer risk (TECR), while the hazard index (HI) for non-carcinogenic effects was obtained by summing the individual HQ values, as expressed in Equations (7) and (8), respectively. Risk estimates were interpreted using a TECR benchmark of 1 × 10^−6^ for carcinogenic effects and an HI threshold of 1 for non-carcinogenic effects [21].

(7) TECR=∑ECR

(8) HI=∑HQ

### 2.4. Source-Oriented Health Risk Assessment of VOCs

Source-oriented health risks were evaluated by integrating the PMF source apportionment results obtained from the VOC dataset with the health risk assessment framework described above. The procedures used to derive the Korea-specific source categories and the corresponding PMF results are described in detailed in the Supplementary Materials.

Importantly, PRTR data were used as supplementary local emission information rather than as a direct quantitative constraint for PMF source assignment. Each PMF factor was first interpreted based on its characteristic VOC profile, the US EPA SPECIATE database, and previously reported source profiles. The chemical characteristics of the resolved factors were then compared with the VOC species and emission characteristics of major PRTR-reported industrial sectors located within approximately 5 km of each monitoring sites. Accordingly, the resulting Korean-specific source categories represent refined source interpretations supported by local emission information rather than direct one-to-one quantitative attribution of individual PMF factors to specific industrial facilities or sectors. Detailed evidence supporting the refinement of individual factors is provided in Table S3.

The primary source-oriented assessment was conducted for adults (19–64 years) under the CTE scenario to characterize source-specific health risks and establish emission-source priorities. For each PMF factor, compound-specific ECR and HQ values were calculated from the apportioned VOC concentrations. The resulting ECR and HQ values were then aggregated within each factor to obtain source-specific TECR and HI, respectively. Concentration-based source contributions from the PMF analysis were then compared with the corresponding health risk-based contributions to identify emission sources and VOC species warranting priority management. To examine whether the source priorities identified for adults were consistent across different life stages, the source-oriented assessment was additionally performed for all age groups using the age-specific exposure factors described above. Detailed results for each age group are presented in Tables S22–S25.

## 3. Results and Discussion

### 3.1. Inhalation Health Risk Assessment of VOCs

**Figure 2:**
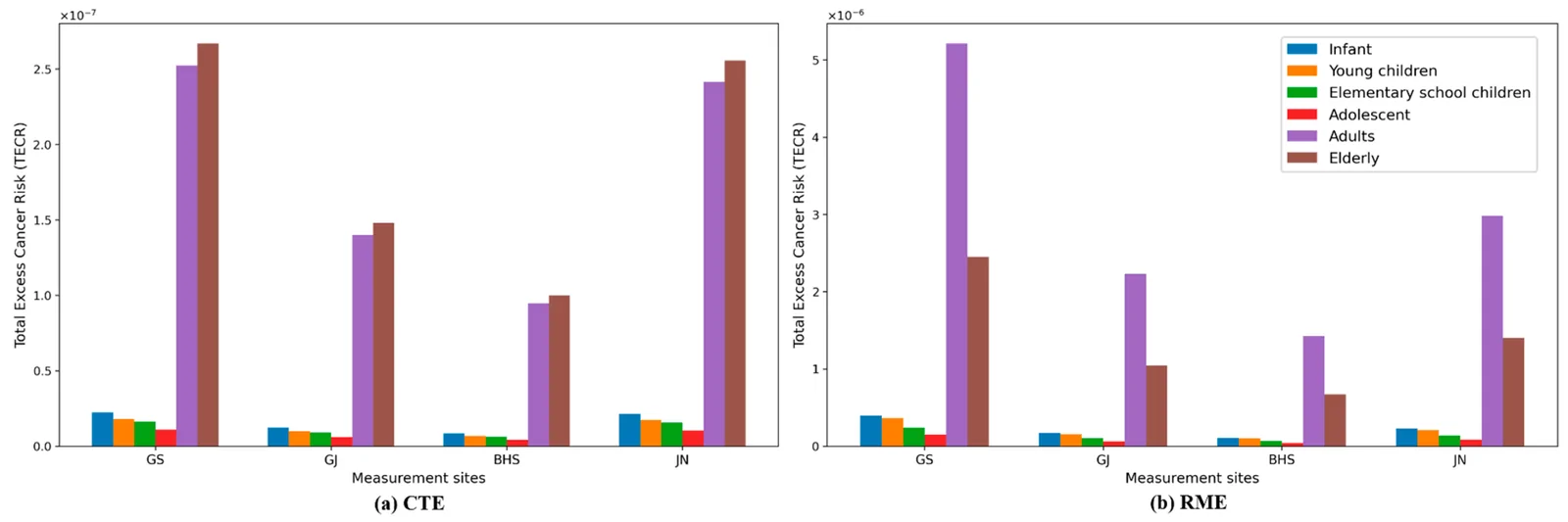
Results of TECR by age group at each measurement site. Results at the CTE level (**a**) results at the RME level (**b**).

**Figure 3:**
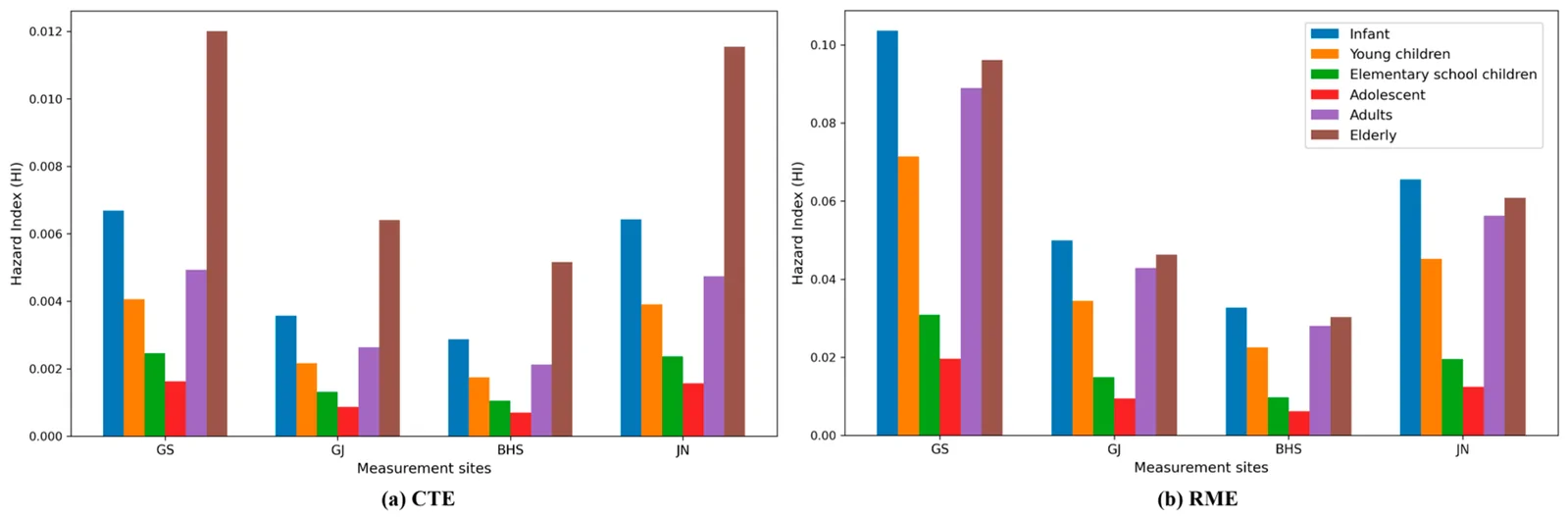
Results of HI by age group at each measurement site. Results at the CTE level (**a**) results at the RME level (**b**).

Prior to the health risk assessment, the concentrations of the 16 selected VOCs were examined to characterize their observed concentration ranges. The concentrations varied considerably among compounds and measurement sites, ranging from 0.02 to 1435.23 ppb at GS, 0.01 to 267.38 ppb at GJ, 0.01 to 68.15 ppb at BHS, and 0.01 to 380.75 ppb at JN (Table S26). Age-specific inhalation risk estimates are presented in Table 3 and Table 4 and Figure 2 and Figure 3, while compound-level results for each age group are provided in Tables S10–S21. The carcinogenic risk assessment results showed that, under the CTE scenario, the TECR values for all measurement sites and age groups did not exceed the acceptable criterion of 1 × 10^−6^, indicating no significant potential for adverse carcinogenic effects. However, under the RME scenario, TECR values exceeded the acceptable criterion of 1 × 10^−6^ for adults, and elderly populations at the GS, GJ, and JN sites, as well as for adults at the BHS site, suggesting the potential occurrence of carcinogenic health effects under prolonged VOC exposure conditions [22]. The relatively higher TECR values observed for adults and the elderly should be interpreted in the context of the age-specific exposure framework applied in this study. In particular, TECR was calculated as a lifetime-averaged risk for exposure occurring over each predefined age interval; therefore, age groups with longer exposure durations can yield higher cumulative lifetime-averaged doses. Accordingly, the higher TECR values for adults and the elderly do not imply that exposure at older ages is intrinsically more carcinogenic than exposure during early life. Rather, these differences primarily reflect the age-specific exposure parameters applied in the assessment, including ED, IR, and BW. The present assessment did not incorporate additional age-dependent susceptibility adjustment factors for early life exposure; therefore, the age-specific TECR values should be interpreted as exposure-based estimates rather than direct measures of age-dependent biological susceptibility to carcinogenesis [23,24].

The compound-specific cancer risk assessment showed that ECR of Ethylbenzene was higher than that of Benzene at the GS, GJ, and JN sites. Although Benzene has traditionally been recognized as a representative carcinogenic VOC and has therefore received considerable attention in air quality management, the relatively higher ambient concentration of Ethylbenzene in this study resulted in a greater contribution to TECR. Recent studies have also identified Ethylbenzene, together with Benzene, as one of the major contributors to cancer risk associated with ambient VOC exposure [25,26,27]. In addition, Ethylbenzene is a representative aromatic VOC that is continuously emitted from various industrial activities, including solvent use, coating processes, printing, and manufacturing. In urban areas such as Seoul, its ambient concentration may be influenced not only by traffic emissions but also by industrial and commercial activities. In contrast, previous studies have reported a general decline in ambient Benzene concentrations in urban areas due to improvements in fuel quality and the strengthening of vehicle emission regulations [3,4,14]. These differences in emission characteristics may have contributed to the relatively greater health risk contribution of Ethylbenzene observed in this study. However, at the BHS site, the ECR of Benzene was higher than that of Ethylbenzene, unlike the other measurement sites. This finding is likely attributable to the distinct VOC composition at the BHS site and suggests that local emission characteristics may influence the predominance of carcinogenic VOCs contributing to cancer risk.

The non-carcinogenic risk assessment showed that HI for all measurement sites and age groups remained below the acceptable threshold of 1 under the CTE scenario, indicating no potential for adverse non-carcinogenic health effects. Similarly, under the RME scenario, HI values at all measurement sites and across all age groups also remained below the threshold of 1, although they were consistently higher than those under the CTE scenario. Compound-specific analysis revealed that Ethylbenzene, Benzene, and Toluene were the primary contributors to the increase in HI. Both the carcinogenic and non-carcinogenic risk assessments consistently identified aromatic compounds as the dominant contributors to health risks, suggesting that they are key target compounds for VOC management in Seoul [4,26]. Therefore, future VOC management strategies should prioritize reducing emissions of aromatic compounds to effectively mitigate potential health risks.

The study period (2018–2024) encompassed the COVID-19 pandemic, during which reductions in traffic volume, changes in industrial activities, and restrictions on human mobility may have temporarily influenced ambient VOC concentrations and source contributions. Although the use of a long-term dataset helped reduce the influence of short-term anomalies, the potential effects of pandemic-related changes in emission characteristics on the health risk assessment results cannot be ruled out. Future studies should distinguish between the pre-pandemic and post-pandemic periods to investigate changes in health risks and source contributions, thereby providing a more robust interpretation of the long-term impacts of variations in emission characteristics.

To evaluate the robustness of the health risk estimates with respect to the 50% detection frequency criterion, an additional MDL-based sensitivity analysis was conducted for the excluded VOC species with applicable inhalation toxicity reference values. Under the toxicity-data framework applied in this study, no additional quantitative ECR could be estimated for the excluded VOCs; consequently, the sensitivity TECR estimates remained identical to the baseline estimates. In contrast, the inclusion of the excluded VOCs increased HI by 36.12~77.58% across the measurement sites, age groups, and exposure scenarios. Nevertheless, all sensitivity HI estimates remained below the acceptable criterion of 1, with the highest sensitivity HI being 0.184. Thus, although the 50% detection frequency criterion may lead to some underestimation of cumulative non-carcinogenic health risks, the conservative MDL-based sensitivity analysis did not alter the overall health risk interpretation of this study. Detailed results are provided in Tables S5–S9.

### 3.2. Source-Oriented Health Risk Assessment of VOCs

Source-specific TECR and HI estimates are presented in Table 5, while the rankings based on concentration and health risk contributions are compared in Table 6 and Figures S5–S8. Source-specific HI values remained below the threshold of 1 for all factors at every measurement site. Similarly, TECR values for all source factors remained below the acceptable cancer risk level of 1 × 10^−6^. These results suggest that individual emission sources or specific industrial activities alone were not sufficient to produce unacceptable health risk under the exposure conditions considered in this study.

Comparison of the two ranking approaches showed that sources contributing substantially to ambient VOC concentrations were not necessarily the dominant contributors to health risk. At the GS site, ‘Metal processing product manufacturing’ ranked first in terms of source contribution; however, its ranking substantially decreased to one of the lowest positions when evaluated based on TECR and HI contribution. In contrast, ‘Other product manufacturing’ ranked third in terms of source contribution but was identified as the highest-ranking source in both TECR- and HI-based contribution. This discrepancy indicates that management priorities should consider the toxicity of emitted VOCs in addition to their concentration contributions.

A comparable shift in source ranking was observed at GJ. ‘Fuel-related emissions’ was the dominant source based on concentration contribution, whereas ‘Metal processing products, chemicals and chemical products manufacturing’ ranked highest when contributions were evaluated using TECR and HI. In addition, ‘Textile product manufacturing; excluding clothing’ ranked relatively low in concentration contribution but became the second-highest contributor when ranked by health risk. This pattern highlights the importance of industrial and manufacturing-related sources when management priorities are established on the basis of health risk rather than VOC concentration alone.

At the JN site, ‘Vehicle exhaust; Acetylene-rich’ was the largest contributor to TECR, whereas ‘Textile product manufacturing; including clothing’ contributed most strongly to HI. Although ‘Background hydrocarbons’ had the highest concentration contribution, it ranked lowest when evaluated on the basis of health risk. These results indicate that traffic- and textile-related sources were more relevant to health risk than their concentration contributions alone world suggest.

At the BHS site, the leading source differed between the two health risk metrics: ‘Chemicals and chemical products manufacturing’ contributed most strongly to TECR, whereas ‘Background hydrocarbons’ was the largest contributor to HI.

Take together, the results for GS, GJ, and JN indicate that VOC management should prioritize emission sources according to their health risk contributions rather than concentration contributions alone. For the background BHS site, however, both local emissions and regional transport may influence health risk, suggesting that management strategies should account for source contributions across a broader spatial scale [3].

To determine whether the source-priority patterns identified for adults were consistent across life stages, the analysis was extended to all age groups (Tables S22–S25). Although absolute source-specific TECR and HI values varied with age-specific exposure factors, the relative rankings of health risk contributions remained unchanged across age groups. In particular, the sources contributing most strongly to TECR and HI in adults retained the same rankings in the other age groups at each measurement site. These results demonstrate that the source-priority patterns identified in the adult-based assessment were robust across life stages.

These findings are consistent with trends reported in previous domestic and international studies. Kwon et al. [28] showed that PMF-based source contribution rankings in Seoul did not necessarily correspond to rankings based on health impacts. Similarly, Eun et al. [4] emphasized the need to consider VOC reactivity, source contributions, and health risks simultaneously when developing urban VOC management strategies. International studies have reported comparable patterns [12,29]. For example, Yang et al. [7] found that although ‘Vehicle exhaust’ ranked first in concentration contribution, ‘Solvent utilization’ was the largest contributor to carcinogenic risk. Likewise, Wang et al. [2] reported that Traffic dominated the concentration contribution, whereas Biogenic sources ranked first for non-carcinogenic risk. These observations support the distinction between concentration-based and health risk-based source priorities identified in the present study.

Overall, the findings support prioritizing manufacturing-related sources in Seoul’s VOC management framework. Nevertheless, source-control strategies should also reflect site-specific emission characteristics: traffic-related influences are particularly relevant at JN, whereas regional transport plays a greater role at the BHS background site. In parallel, Benzene, Ethylbenzene, and Toluene, which were identified as major contributors to health risk, should receive particular attention in compound-specific emission reduction efforts.

From a management perspective, the identified manufacturing-related sources should be treated as priority sectors for further process-level investigation rather than as uniform targets for sector-wide control. Future efforts should therefore focus on identifying specific VOC-emitting processes within these sectors, particularly those associated with Benzene, Ethylbenzene, and Toluene emissions. However, the PMF and PRTR data used in this study do not provide sufficient process-level resolution to quantitatively distinguish individual manufacturing processes within each industrial category. Therefore, further integration of facility- and process-specific emission information is needed to develop more targeted VOC control strategies.

Furthermore, the consistency of source-specific risk rankings across age groups indicates that the identified priority sources were not dependent on the selection of adults as the representative population. Therefore, although absolute health risk levels differed among age groups, the source-oriented management priorities derived in this study were consistently applicable to sensitive populations, including infants, children, and the elderly.

The PMF-derived source factors used in this study were interpreted by integrating statistically resolved factor profiles with the previous literature and PRTR data. However, PRTR data represent facility-reported emission quantities, whereas PMF factors are derived from temporal variations in ambient concentrations measured at receptor sites. Therefore, differences in temporal resolution, spatial representativeness, atmospheric transport and transformation, and chemical composition may limit the direct comparability of the two datasets. Accordingly, PRTR information was used only as supplementary local evidence for refining the interpretation of PMF-resolved source profiles and should not be interpreted as a direct quantitative attribution of individual PMF factors to specific industrial facilities or sectors. Accordingly, each factor should be regarded as representing a mixture of emission sources with similar chemical characteristics rather than a single, well-defined emission source. Moreover, because different emission sources can exhibit similar VOC compositions, uncertainty in factor interpretation is unavoidable. Although Bootstrap and DISP analyses were not performed in the present study, these approaches could provide additional information on factor stability and rotational ambiguity, respectively. Future studies should therefore improve the robustness of source identification by incorporating these PMF uncertainty analyses together with additional emission inventory data [30,31,32].

In addition, the health risk estimates in this study were derived using a deterministic approach based on CTE and RME scenarios and therefore do not represent probabilistic confidence intervals. Although the use of CTE and RME scenarios allowed health risks to be evaluated under both typical and higher-exposure conditions, uncertainties associated with exposure parameters, toxicity values, and concentration variability were not probabilistically quantified. Future studies incorporating probabilistic approaches, such as Monte Carlo simulation, could provide a more comprehensive characterization of uncertainty and confidence intervals for the estimated health risks.

Although pulmonary uptake may vary among individual VOC species, compound-specific absorption fractions were not separately applied because the inhalation toxicity values used in this study were derived specifically for the inhalation pathway. Nevertheless, inter-compound difference in pulmonary uptake may contribute to residual uncertainty in the estimated relative health risk contributions.

In addition, to ensure data reliability, the primary health risk assessment was based on 16 VOC species with detection frequencies greater than 50%. Because this selection criterion may exclude compounds with lower detection frequencies but potentially relevant toxicological effects, an additional MDL-based sensitivity analysis was conducted. The results showed that inclusion of the excluded VOCs with applicable inhalation toxicity reference values increased HI by 36.12–77.58%, although all sensitivity HI estimates remained below 1 and the overall non-carcinogenic risk interpretation remained unchanged. No additional quantitative ECR could be estimated for the excluded VOCs under the toxicity-data framework applied in this study; therefore, the TECR estimates remained unchanged.

Nevertheless, the sensitivity analysis does not account for hazardous VOCs that were not included in the original long-term PAMS dataset. Several highly toxic VOCs, including formaldehyde, 1,3-butadiene, and acrolein, could not be evaluated because consistent long-term monitoring data covering the study period were unavailable. Therefore, although the sensitivity analysis supports the robustness of the principal health risk interpretation with respect to the 50% detection frequency criterion, the health risks reported in this study should not be interpreted as representing the complete health burden associated with all hazardous VOCs in ambient air. Future studies incorporating a broader range of hazardous VOCs with consistent long-term monitoring data are warranted to provide a more comprehensive assessment of cumulative health risks.

## 4. Conclusions

This study evaluated age-specific and source-oriented health risks of VOCs at four measurement sites in Seoul (GS, GJ, BHS, and JN) to identify priority emission sources and compounds by comparing concentration-based and health risk-based source contributions. Under the CTE scenario, TECR and HI remained below the acceptable criteria at all measurement sites. Under the RME scenario, however, TECR exceeded the acceptable criterion of 1 × 10^−6^ for the adult and elderly age groups, indicating the potential for increased health risks under higher-exposure conditions. In addition, the compound-specific health risk assessment revealed that Benzene and Ethylbenzene were the major contributors to TECR. Except for the BHS site, the health impact contribution of Ethylbenzene was greater than that of Benzene at all measurement sites. Furthermore, Ethylbenzene, Benzene, and Toluene were identified as the major contributors to HI.

The source-based health risk assessment showed that concentration-based and health risk-based source contributions differed across the identified emission sources. At GS, ‘Metal processing product manufacturing’ ranked first in concentration contribution, whereas ‘Other product manufacturing’ was the largest contributor to health risk. At GJ, ‘Fuel-related emissions’ had the highest concentration contribution, while ‘Metal processing products, chemicals and chemical products manufacturing’ contributed most strongly to health risk. At BHS, ‘Background hydrocarbons’ ranked first in concentration contribution, whereas ‘Chemicals and chemical products manufacturing’ ranked relatively higher based on health risk. At JN, despite the dominant concentration contribution of ‘Background hydrocarbons’, ‘Vehicle exhaust: Acetylene-rich’ and ‘Textile product manufacturing’ were identified as major health risk contributors. These findings demonstrate that the sources dominating ambient VOC concentrations are not necessarily those contributing most strongly to health risk. Accordingly, the results suggest that manufacturing-related emission sources should be considered priority management targets in the establishment of VOC reduction strategies for Seoul. For practical management, these priority manufacturing categories should be further examined at the process level to identify specific VOC-emitting activities associated with Benzene, Ethylbenzene, and Toluene. Because the present PMF-PRTR framework does not provide sufficient resolution for quantitative process-level attribution, future studies integrating detailed facility- and process-specific emission information are needed to develop more targeted control strategies.

This study extended beyond conventional concentration-based VOC assessments by integrating source apportionment results with health risk assessments to evaluate source-specific health impact characteristics. Furthermore, by comparing source contribution estimates with health risk-based contributions, the study identified priority emission sources and compounds from the perspective of actual health impacts. This integrated approach is expected to provide fundamental scientific information for the development of health impact-based management strategies, rather than simple concentration reduction approaches, in future urban VOC management policies.

## Figures and Tables

**Table 2:** Exposure factor of this study.

Exposure Factor	Age Group	CTE	RME	Reference
Body Weight (BW, kg)	Infant (0–2 years)	8.7		[17,18]
	Young children (3–6 years)	18.8		
	Elementary school children (7–12 years)	36.9		
	Adolescent (13–18 years)	59.9		
	Adults (19–64 years)	65.3		
	Elderly (65–84 years)	58.9		
Inhalation Rate (IR, m^3^/day)	Infant (0–2 years)	9.49	10.2	[17,18]
	Young children (3–6 years)	10.38	12.02	
	Elementary school children (7–12 years)	11.84	13.32	
	Adolescent (13–18 years)	14.68	17.75	
	Adults (19–64 years)	14.60	18.25	
	Elderly (65–84 years)	14.60	18.25	
Exposure Time (ET, h/day)	Infant (0–2 years)	0.60	1.63	[17,18]
	Young children (3–6 years)	0.72	2.06	
	Elementary school children (7–12 years)	0.75	1.58	
	Adolescent (13–18 years)	0.65	1.22	
	Adults (19–64 years)	2.16	5.87	
	Elderly (65–84 years)	4.74	5.72	
Exposure Frequency (EF, day/yr)	All ages	300	330	[15]
Exposure Duration (ED, yr)	Infant (0–2 years)	3		This Study
	Young children (3–6 years)	4		
	Elementary school children (7–12 years)	6		
	Adolescent (13–18 years)	6		
	Adults (19–64 years)	46		
	Elderly (65–84 years)	20		
Average Time (AT, h)	All ages	ED × 365 × 24		
Lifetime (LT, h)	All ages	84 × 365 × 24		[19]

**Table 3:** Results of TECR by age group at each measurement site.

Age Group	Measurement Site							
	GS		GJ		BHS		JN	
	CTE ^a^	RME ^b^	CTE	RME	CTE	RME	CTE	RME
Infant	2.23 × 10^−8^	3.96 × 10^−7^	1.24 × 10^−8^	1.69 × 10^−7^	8.36 × 10^−9^	1.08 × 10^−7^	2.13 × 10^−8^	2.26 × 10^−7^
Young children	1.81 × 10^−8^	3.64 × 10^−7^	1.00 × 10^−8^	1.56 × 10^−7^	6.77 × 10^−9^	9.96 × 10^−8^	1.73 × 10^−8^	2.08 × 10^−7^
Elementary school children	1.64 × 10^−8^	2.36 × 10^−7^	9.09 × 10^−9^	1.01 × 10^−7^	6.15 × 10^−9^	6.47 × 10^−8^	1.57 × 10^−8^	1.35 × 10^−7^
Adolescent	1.09 × 10^−8^	1.50 × 10^−7^	6.02 × 10^−9^	6.41 × 10^−8^	4.07 × 10^−9^	4.10 × 10^−8^	1.04 × 10^−8^	8.57 × 10^−8^
Adults	2.52 × 10^−7^	5.21 × 10^−6^	1.40 × 10^−7^	2.23 × 10^−6^	9.46 × 10^−8^	1.43 × 10^−6^	2.41 × 10^−7^	2.98 × 10^−6^
Elderly	2.67 × 10^−7^	2.45 × 10^−6^	1.48 × 10^−7^	1.05 × 10^−6^	1.00 × 10^−7^	6.70 × 10^−7^	2.55 × 10^−7^	1.40 × 10^−6^

^a^ CTE: central tendency exposure. ^b^ RME: reasonable maximum exposure.

**Table 4:** Results of HI by age group at each measurement site.

Age Group	Measurement Site							
	GS		GJ		BHS		JN	
	CTE	RME	CTE	RME	CTE	RME	CTE	RME
Infant	6.69 × 10^−3^	1.04 × 10^−1^	3.57 × 10^−3^	4.99 × 10^−2^	2.87 × 10^−3^	3.27 × 10^−2^	6.43 × 10^−3^	6.56 × 10^−2^
Young children	4.06 × 10^−3^	7.14 × 10^−2^	2.17 × 10^−3^	3.44 × 10^−2^	1.75 × 10^−3^	2.25 × 10^−2^	3.90 × 10^−3^	4.52 × 10^−2^
Elementary school children	2.46 × 10^−3^	3.09 × 10^−2^	1.31 × 10^−3^	1.49 × 10^−2^	1.06 × 10^−3^	9.75 × 10^−3^	2.36 × 10^−3^	1.96 × 10^−2^
Adolescent	1.63 × 10^−3^	1.96 × 10^−2^	8.68 × 10^−4^	9.45 × 10^−3^	6.99 × 10^−4^	6.18 × 10^−3^	1.56 × 10^−3^	1.24 × 10^−2^
Adults	4.93 × 10^−3^	8.90 × 10^−2^	2.63 × 10^−3^	4.29 × 10^−2^	2.12 × 10^−3^	2.80 × 10^−2^	4.74 × 10^−3^	5.63 × 10^−2^
Elderly	1.20 × 10^−2^	9.61 × 10^−2^	6.41 × 10^−3^	4.63 × 10^−2^	5.16 × 10^−3^	3.03 × 10^−2^	1.15 × 10^−2^	6.08 × 10^−2^

**Table 5:** Results of TECR and HI by source factors at each measurement site.

Source Factors	Measurement Site							
	GS		GJ		BHS		JN	
	TECR ^a^	HI ^b^	TECR	HI	TECR	HI	TECR	HI
Factor 1	6.52 × 10^−8^	1.74 × 10^−3^	1.19 × 10^−7^	1.76 × 10^−3^	3.89 × 10^−8^	5.13 × 10^−4^	8.62 × 10^−9^	1.59 × 10^−4^
Factor 2	5.16 × 10^−10^	6.93 × 10^−5^	9.82 × 10^−9^	2.88 × 10^−4^	3.04 × 10^−8^	8.77 × 10^−4^	1.69 × 10^−8^	2.12 × 10^−4^
Factor 3	2.73 × 10^−8^	5.11 × 10^−4^	5.07 × 10^−8^	1.29 × 10^−3^	4.52 × 10^−8^	7.91 × 10^−4^	1.29 × 10^−7^	2.04 × 10^−3^
Factor 4	2.91 × 10^−7^	4.54 × 10^−3^	5.56 × 10^−9^	1.20 × 10^−4^	2.52 × 10^−8^	7.52 × 10^−4^	7.91 × 10^−8^	2.29 × 10^−3^
Factor 5	3.61 × 10^−11^	1.06 × 10^−5^	1.50 × 10^−8^	2.90 × 10^−4^	-	-	1.41 × 10^−8^	2.82 × 10^−4^
Factor 6	1.05 × 10^−8^	4.74 × 10^−4^	2.31 × 10^−9^	1.07 × 10^−4^	-	-	4.66 × 10^−8^	7.38 × 10^−4^

^a^ TECR: total excess cancer risk. ^b^ HI: hazard index.

**Table 6:** Comparison of source contribution and risk-based contribution of VOC sources.

Measurement Site	Source Contribution	Risk-Based Contribution (TECR)	Risk-Based Contribution (HI)
GS	Metal processing product manufacturing (41.1%) > Vehicle exhaust (15.9%) > Other product manufacturing (12.7%) > LPG use (11.8%) > Gasoline evaporation (10.5%) > Industrial emissions (7.7%)	Other product manufacturing (73.8%) > Vehicle exhaust (16.5%) > Gasoline evaporation (6.9%) > Industrial emissions (2.7%) > LPG use (0.1%) > Metal processing product manufacturing (0.01%)	Other product manufacturing (61.8%) > Vehicle exhaust (23.7%) > Gasoline evaporation (7.0%) > Industrial emissions (6.5%) > LPG use (0.9%) > Metal processing product manufacturing (0.1%)
GJ	Fuel-related emissions (32.6%) > Vehicle exhaust (21.4%) > Metal processing products, chemicals and chemical products manufacturing (17.0%) > Gasoline evaporation (16.0%) > Textile product manufacturing; excluding clothing (7.5%) > LPG use (5.6%)	Metal processing products, chemicals and chemical products manufacturing (58.8%) > Textile product manufacturing; excluding clothing (25.1%) > Fuel-related emissions (7.4%) > Vehicle exhaust (4.9%) > LPG use (2.7%) > Gasoline evaporation (1.1%)	Metal processing products, chemicals and chemical products manufacturing (45.7%) > Textile product manufacturing; excluding clothing (33.5%) > Fuel-related emissions (7.5%) > Vehicle exhaust (7.5%) > LPG use (3.1%) > Gasoline evaporation (2.8%)
BHS	Background hydrocarbons (33.8%) > Gasoline evaporation (25.9%) > Textile product manufacturing; excluding clothing (23.0%) > Chemicals and chemical products manufacturing (17.3%)	Chemicals and chemical products manufacturing (32.4%) > Textile product manufacturing; excluding clothing (27.8%) > Background hydrocarbons (21.8%) > Gasoline evaporation (18.0%)	Background hydrocarbons (29.9%) > Chemicals and chemical products manufacturing (27.0%) > Gasoline evaporation (25.6%) > Textile product manufacturing; excluding clothing (17.5%)
JN	Background hydrocarbons (28.9%) > Fuel-related emissions (22.0%) > Vehicle exhaust: Acetylene-rich (16.0%) > Textile product manufacturing; including clothing (15.9%) > Vehicle exhaust: Benzene-rich (9.7%) > Chemicals and chemical products manufacturing (7.4%)	Vehicle exhaust: Acetylene-rich (43.8%) > Textile product manufacturing; including clothing (26.9%) > Chemicals and chemical products manufacturing (15.8%) > Fuel-related emissions (5.7%) > Vehicle exhaust: Benzene-rich (4.8%) > Background hydrocarbons (2.9%)	Textile product manufacturing; including clothing (40.0%) > Vehicle exhaust: Acetylene-rich (35.7%) > Chemicals and chemical products manufacturing (12.9%) > Vehicle exhaust: Benzene-rich (4.9%) > Fuel-related emissions (3.7%) > Background hydrocarbons (2.8%)

## Data Availability

The data supporting the findings of this study were provided by the SIHE. The data are not publicly available due to restrictions imposed by the data provider and cannot be shared by the authors.

## Supplementary Materials

The following supporting information can be downloaded at https://www.mdpi.com/article/10.3390/toxics14090799/s1. Figure S1: Refined PMF source profiles at the GS based on PRTR emission linkage; Figure S2: Refined PMF source profiles at the GJ based on PRTR emission linkage; Figure S3: Refined PMF source profiles at the BHS based on PRTR emission linkage; Figure S4: Refined PMF source profiles at the JN based on PRTR emission linkage; Figure S5: Comparison of source and risk-based contribution rankings of VOC sources at GS; Figure S6: Comparison of source and risk-based contribution rankings of VOC sources at GJ; Figure S7: Comparison of source and risk-based contribution rankings of VOC sources at BHS; Figure S8: Comparison of source and risk-based contribution rankings of VOC sources at JN; Table S1: Measurement and cut-off ranges of valid observation; Table S2: Method detection limits for VOCs by measurement sites (unit: ppb); Table S3: Comparison of PMF source profiles and PRTR emission characteristics used for source reinterpretation; Table S4: Detection frequency of 56 types of VOCs by measurement sites; Table S5: toxicity information of VOC species included in the MDL-based sensitivity analysis; Table S6: Comparison of baseline and MDL-based sensitivity estimates of TECR and HI by age group at the GS site; Table S7: Comparison of baseline and MDL-based sensitivity estimates of TECR and HI by age group at the GJ site; Table S8: Comparison of baseline and MDL-based sensitivity estimates of TECR and HI by age group at the BHS site; Table S9: Comparison of baseline and MDL-based sensitivity estimates of TECR and HI by age group at the JN site; Table S10: Results of ECR for individual VOCs at each measurement site (infant); Table S11: Results of ECR for individual VOCs at each measurement site (young children); Table S12: Results of ECR for individual VOCs at each measurement site (elementary school children); Table S13: Results of ECR for individual VOCs at each measurement site (adolescent); Table S14: Results of ECR for individual VOCs at each measurement site (adults); Table S15: Results of ECR for individual VOCs at each measurement site (elderly); Table S16: Results of HQ for individual VOCs at each measurement site (infant); Table S17: Results of HQ for individual VOCs at each measurement site (young children); Table S18: Results of HQ for individual VOCs at each measurement site (elementary school children); Table S19: Results of HQ for individual VOCs at each measurement site (adolescent); Table S20: Results of HQ for individual VOCs at each measurement site (adults); Table S21: Results of HQ for individual VOCs at each measurement site (elderly); Table S22: Age-specific source-oriented health risk rankings at the GS site; Table S23: Age-specific source-oriented health risk rankings at the GJ site; Table S24: Age-specific source-oriented health risk rankings at the BHS site; Table S25: Age-specific source-oriented health risk rankings at the JN site; Table S26: Descriptive statistics of the concentrations of the 16 selected VOCs at each measurement site [33,34].
