# Supplementary material for: Selection of Priority VOC Emission Sources in Seoul by Comparing Source Contributions and Health Risk-Based Contributions
Source: Toxics. 2026 Sep 9;14(9):799. doi: 10.3390/toxics14090799 (PMC13611053; doi:10.3390/toxics14090799)
Supplement: Supplementary file 1 [file toxics-14-00799-s001.zip › toxics-4518826-supplementary.pdf]

toxics

MDPI

Article

Selection of Priority VOC Emission Sources in Seoul by Comparing Source Contributions and Health Risk-based Contributions

Ji-Yun Jung [1], Shin-Young Park [1], Dong-Seop Shin [1], Jong-Cheol Yoon [2], Hak-Myeong Lim [2], Kwang-Rae Kim [2], Seok-Ryul Oh [2], Yong-Suk Choi [2], Cheol-Min Lee [1,*]

[1] Department of Chemical and Environmental Engineering, Seokyeong University, Seoul 02713, Republic of Korea; jju1049@skuniv.ac.kr (J.-Y.J.); tlsdud060900@skuniv.ac.kr (S.-Y.P.); sds920703@gmail.com (D.-S.S.)

[2] Seoul Metropolitan Government Research Institute of Public Health and Environment, Seoul 13818, Republic of Korea; yoonkjc@seoul.go.kr (J.-C.Y); chayke1@seoul.go.kr (H.-M.L); kimkr@seoul.go.kr (K.-R.K); ifnwu@seoul.go.kr (S.-R.O.); hozer87@seoul.go.kr (Y.-S.C.)

* Correspondence: cheolmin@skuniv.ac.kr

Abstract: This study conducted age-specific and source-oriented health risk assessments of volatile organic compounds (VOCs) at four measurement sites in Seoul (GS, GJ, BHS, and JN). By comparing source-specific concentration contributions with health risk-based contributions, priority sources and compounds were identified. The age-specific health risk assessment showed that, under the central tendency exposure (CTE) scenario, both the total excess cancer risk (TECR) and hazard index (HI) at all measurement sites remained below the recommended guideline values. However, under the reasonable maximum exposure (RME) scenario, TECR exceeded 1×10^{-6} for some adult and elderly groups, indicating the potential for adverse health effects. Compound-specific analysis identified Benzene and Ethylbenzene as the major contributors to TECR, whereas Ethylbenzene, Benzene, and Toluene were the primary contributors to HI. The source-oriented health risk assessment revealed that concentration-based and health risk-based source contributions exhibited different patterns, with manufacturing-related sources ranking among the highest contributors to health risks at all measurement sites. At the GS site, the highest-ranking sources in terms of concentration and health risk contributions were *Metal processing product manufacturing* and *Other product manufacturing*, respectively. At the GJ site, *Fuel-related emissions* ranked highest in concentration contribution, whereas *Metal processing products, chemicals and chemical products manufacturing* ranked highest in health risk contribution. At the BHS and JN sites, *Background hydrocarbons* showed the greatest concentration contribution, while *Chemicals and chemical products manufacturing, Vehicle exhaust: Acetylene-rich,* and *Textile product manufacturing* were the major contributors. This study demonstrates that VOC management in Seoul should prioritize health risk-based rather than concentration-based approaches.

Keywords: Volatile Organic Compounds; Health Risk Assessment; Source-oriented Risk Assessment, Source Contribution, Risk-based Source Prioritization

1. Measurement and Analysis of VOCs

Volatile organic compounds (VOCs) were measured using the Photochemical Assessment Monitoring Stations (PAMSs) operated by the Seoul Metropolitan Government Research Institute of Health and Environment. The monitoring was conducted in accordance with Article 3 (Continuous Monitoring) and Article 11 (Establishment of Comprehensive Air Quality Improvement Plans) of the Clean Air Conservation Act, as well as the Guidelines for Installation and Operation of Air Pollution Monitoring Networks [32,33].

 https://doi.org/10.3390/toxics14090799

The VOCs measured at the PAMS sites were analyzed in real time according to the "Automatic Measurement Method for Ozone Precursors in Ambient Air (ES 01805.1)" specified in the Korean Standard Methods for Air Pollution [32]. Ambient air was directly introduced into the online automated GC/FID system using a miniature pump at a flow rate of 15 mL/min for 40 min, from 10 to 50 min past each hour, resulting in a total sampling volume of approximately 600 mL. Because an online automated sampling system was used, separate offline adsorbent sampling tubes were not employed. Instead, the sampled air was dried using a Nafion dryer and subsequently concentrated in a low-temperature concentration trap incorporated in the automated thermal desorption system. At the end of each sampling period, the concentrated VOCs were thermally desorbed and transferred to the GC system for separation and FID detection.

The GC system employed two analytical columns according to the molecular weight range of the target VOCs: a PLOT column (0.32 mm, 50 m, 5 μm) for relatively light hydrocarbons and a BP-1 column (0.22 mm, 50 m, 1 μm) for relatively heavier hydrocarbons. The thermal desorption and GC operating conditions included a sampling flow rate of 15 mL/min, a sampling time of 40 min, a thermal-desorber pressure of 39 psi, a tube desorption temperature of 100℃ for 1 min, a valve temperature of 175℃, a transfer-line temperature of 200℃, and an FID temperature of 250℃. The GC oven was initially maintained at 46℃ for 15 min, increased to 170℃ at 5℃/min, and subsequently increased to 200℃ at 15℃/min (Table S1).

Quantification of the target VOCs was performed based on the carbon response of the FID according to the analytical procedure used for the PAMS automated GC/FID system. For relatively light hydrocarbons (C2-C5) separated on the PLOT column, propane was used as the reference compound, whereas Benzene was used as the reference compound for relatively heavier hydrocarbons (C5-C12) separated on the BP-1 column. Calibration was performed using standard gas mixtures, including NIST SRM 1800 and certified reference materials (CRM; SPECTRA), and multi-point calibration was conducted at 1,5, and 10 ppbv. Concentrations expressed on a carbon basis were converted to compound concentrations by accounting for the number of carbon atoms in each target compound. Thus, quantification was based on the carbon-response calibration of the respective analytical channel rather than on independently determined response factors for each individual VOC.

Table S1. Measurement and cut-off ranges of valid observation.

Item	Standard	Test Result	Method
System blank analysis	The sum of total peak < 20ppbv VOCs conc. per each column < 10ppbv VOCs conc.	PLOT: 0.0013<br>BP-1: 0.0028	Use humidified N_2 gas
Multiple points cal. linearity	$R^2 \geq 0.995$	PLOT: 0.9965<br>BP-1: 0.9999	1,5,10 ppbv as propane, benzene, RT < ±0.5min
MDL (Method Detection Limit) test	< 2ppbv as propane, benzene	Propane: 0.39<br>Benzene: 0.49	1 ppbv 7 times measurement in a row
Precision test	Within ±20% RPD	Propane: 1.31<br>Benzene: 0.83	5 ppbv 2 times measurements
Resolution	56 target components	Normal separation	-

https://doi.org/10.3390/toxics14090799

2. Quality Assurance/Quality Control (QA/QC)

Annual quality assurance and quality control (QA/QC) procedures were conducted to ensure the reliability of the analytical results. The evaluated QA/QC parameters included the Method Detection Limit (MDL), relative standard deviation (RSD), and the coefficient of determination (R^2) of the calibration curve. The MDL was calculated by multiplying the standard deviation of seven repeated measurements by 3.14. The RSD (%) was calculated by dividing the standard deviation of repeated measurements of standard materials by the mean value and multiplying by 100; in this study, RSD values were determined based on four repeated measurements. In addition, calibration curves were established using four standard materials within the quantification range, and the linearity of the calibration curves was evaluated using the coefficient of determination (R^2). Calibration curves with R^2 values lower than 0.99 were reconstructed. The QA/QC results obtained in this study demonstrated that all criteria for blank test, R^2, MDL, and precision were satisfied, confirming the reliability of the VOC analytical data (Table S2). The MDLs of the VOC species ranged from 0.013 ppbv for n-Dodecane to 0.119 ppbv for Acetylene, indicating sufficient analytical sensitivity for ambient VOC measurements.

Table S2. Method Detection Limits for VOCs by measurement sites (unit: ppb).

VOCs	Measurement sites			
	GS	GJ	BHS	JN
Ethane	0.0839	0.0766	0.0766	0.0839
Ethylene	0.1083	0.0593	0.0839	0.0839
Propane	0.0996	0.0559	0.0722	0.0396
Propylene	0.0511	0.0559	0.0824	0.0722
i-Butane	0.0383	0.0383	0.0747	0.0747
n-Butane	0.0297	0.0706	0.0890	0.0618
Acetylene	0.1187	0.0593	0.0839	0.0906
trans-2-Butene	0.0420	0.0785	0.0873	0.0641
1-Butene	0.0420	0.0839	0.0706	0.0747
cis-2-Butene	0.0593	0.0453	0.0641	0.0839
Cyclopentane	0.0363	0.0699	0.0565	0.0877
i-Pentane	0.0433	0.0565	0.0597	0.0867
n-Pentane	0.0433	0.0811	0.0867	0.0763
trans-2-Pentene	0.0306	0.0712	0.0919	0.0949
1-Pentene	0.0306	0.0613	0.0867	0.0888
cis-2-Pentene	0.0494	0.0565	0.0494	0.0949
2,2-Dimethylbutane	0.0198	0.0582	0.0722	0.0791
2,3-Dimethylbutane	0.0198	0.0656	0.0704	0.0396
2-Methylpentane	0.0198	0.0471	0.0656	0.0676
3-Methylpentane	0.0427	0.0511	0.0906	0.0899
Isoprene	0.0336	0.0306	0.0867	0.0628
1-Hexane	0.0396	0.0255	0.0906	0.0666
Hexane	0.0511	0.0766	0.0471	0.0899

https://doi.org/10.3390/toxics14090799

Methylcyclopentane	0.0847	0.0666	0.0942	0.0783
2,4-Dimethylpentane	0.0876	0.0438	0.0480	0.0499
Benzene	0.0302	0.0361	0.0656	0.0498
Cyclohexane	0.0731	0.0302	0.0839	0.0892
2-Methylhexane	0.0719	0.0404	0.0404	0.0427
2,3-Dimethylpentane	0.0706	0.0404	0.0438	0.0427
3-Methylhexane	0.0657	0.0545	0.0366	0.0848
2,2,4-Trimethylpentane	0.0902	0.0383	0.0499	0.0717
n-Heptane	0.0240	0.0219	0.0619	0.0562
Methylcyclohexane	0.0819	0.0449	0.0571	0.0771
2,3,4-Trimethylpentane	0.0373	0.0353	0.0353	0.0641
Toluene	0.0427	0.0892	0.0765	0.0771
2-Methylheptane	0.0420	0.0271	0.0373	0.0742
3-Methylheptane	0.0383	0.0420	0.0383	0.0873
n-Octane	0.0445	0.0271	0.0437	0.0926
Ethylbenzene	0.0437	0.0192	0.0210	0.0869
m,p-Xylene	0.0383	0.0618	0.0445	0.0752
Styrene	0.0542	0.0210	0.0600	0.0799
o-Xylene	0.0353	0.0383	0.0210	0.0747
n-Nonane	0.0444	0.0186	0.0241	0.0694
i-Propylbenzene	0.0241	0.0444	0.0264	0.0851
n-Propylbenzene	0.0332	0.0403	0.0264	0.0865
m-Ethyltoluene	0.0275	0.0241	0.0424	0.0698
p-Ethyltoluene	0.0469	0.0275	0.0570	0.0698
1,3,5-Trimethylbenzene	0.0264	0.0264	0.0285	0.0898
o-Ethyltoluene	0.0403	0.0275	0.0332	0.0659
1,2,4-Trimethylbenzene	0.0201	0.0522	0.0186	0.0960
n-Decane	0.0256	0.0433	0.0237	0.0744
1,2,3-Trimethylbenzene	0.0373	0.0373	0.0314	0.0632
m-Diethylbenzene	0.0336	0.0620	0.0460	0.0601
p-Diethylbenzene	0.0247	0.0597	0.0283	0.0613
n-Undecane	0.0347	0.0363	0.0165	0.0930
n-Dodecane	0.0128	0.0255	0.0249	0.0827

3. Refinement of Source Identification through PRTR-based Emission Linkage

The major emission sources of VOCs in Seoul were identified using the US EPA PMF 5.0 model. The PMF analysis was conducted using measured VOC concentration data together with associated uncertainty data. The range of factor numbers was initially determined based on the PCA results, and the final number of factors was selected by comprehensively considering the Q(robust) and Q(true) values, as well as the interpretability of the identified factors. Source interpretation was performed with reference to the US EPA SPECIATE database and previously reported source-specific chemical composition characteristics of VOCs from earlier studies.

To refine the interpretation of the PMF results by incorporating domestic emission characteristics, Pollutant Release and Transfer Register (PRTR) data were used as supplementary local emission information. Atmospheric VOC emissions by industrial sector were investigated for facilities located within an approximately 5 km radius of each measurement site, including their industrial classifications, reported VOC species, and emission amounts. Importantly, PRTR data were not used to directly assign or quantitatively constrain individual PMF factors because PMF factors are derived from temporal variations in ambient concentrations at receptor sites, whereas PRTR data represent facility-reported emission quantities. Instead, each PMF factor was first interpreted based on its dominant and characteristic VOC species, the US EPA SPECIATE database, and previously reported source profiles. The chemical characteristics of each factor were subsequently compared with the VOC species and emission characteristics reported for major nearby PRTR industrial sectors. When consistency was observed between the PMF chemical profile and locally relevant PRTR emission characteristics, the initial source interpretation was refined to reflect domestic emission characteristics. Therefore, the PRTR-based source categories should be interpreted as refined source interpretations supported by local emission information rather than as direct one-to-one quantitative attribution of individual PMF factors to specific PRTR facilities or industrial sectors. The detailed evidence supporting these refined source interpretations is summarized in Table S3.

The source apportionment results for each measurement site refined using PRTR information are presented in Figs. S1-S4. At the GS site, integration with PRTR data led to the reinterpretation of Factor 5, which had previously been identified as 'Fuel storage emissions,' as 'Metal processing product manufacturing,' while Factor 4, previously interpreted as 'Solvent use,' was reinterpreted as 'Other product manufacturing.' At the GJ site, Factor 1, previously identified as 'Solvent use,' was reinterpreted as 'Metal processing products, chemicals and chemical products manufacturing,' whereas Factor 3, previously interpreted as 'Fuel evaporation,' was reinterpreted as 'Textile product manufacturing; excluding clothing.' At the BHS site, Factor 1, previously interpreted as 'Solvent use,' was reinterpreted as 'Textile product manufacturing; excluding clothing,' while Factor 3, previously identified as 'Secondary hydrocarbons,' was reinterpreted as 'Chemicals and chemical products manufacturing.' At the JN site, Factor 3, previously interpreted as 'Solvent use,' was reinterpreted as 'Chemicals and chemical products manufacturing,' whereas Factor 6, previously identified as 'Secondary/aged hydrocarbons,' was reinterpreted as 'Textile product manufacturing; including clothing.'

Table S3. Comparison of PMF source profiles and PRTR emission characteristics used for source reinterpretation.

Measurement Sites	Factor	Initial Interpretation	Characteristic Species in PMF Profile	Supporting PRTR Industrial Sector
GS	Factor 4	Solvent use	Aromatic VOCs, particularly Xylene	Other product manufacturing
	Factor 5	Fuel storage emissions	n-Butane-dominant profile	Metal processing products

https://doi.org/10.3390/toxics14090799

				manufacturing; excluding machinery and furniture
GJ	Factor 1	Solvent use	Toluene, Xylene-rich profile	Metal processing products manufacturing; chemicals and chemical products manufacturing
	Factor 3	Fuel evaporation	Toluene and Hexane	Textile product manufacturing; excluding clothing
BHS	Factor 1	Solvent use	Toluene and Xylene	Textile product manufacturing; excluding clothing
	Factor 3	Secondary hydrocarbons	Toluene, Xylene, and Hexane	Chemicals and chemical products manufacturing; excluding pharmaceuticals
JN	Factor 3	Solvent use	Toluene, Xylene, and Ethylbenzene	Chemicals and chemical products manufacturing; excluding pharmaceuticals
	Factor 6	Secondary/aged hydrocarbons	Toluene, Xylene, and heavier hydrocarbons	Textile product manufacturing; excluding clothing/clothing, clothing accessories, and fur product manufacturing

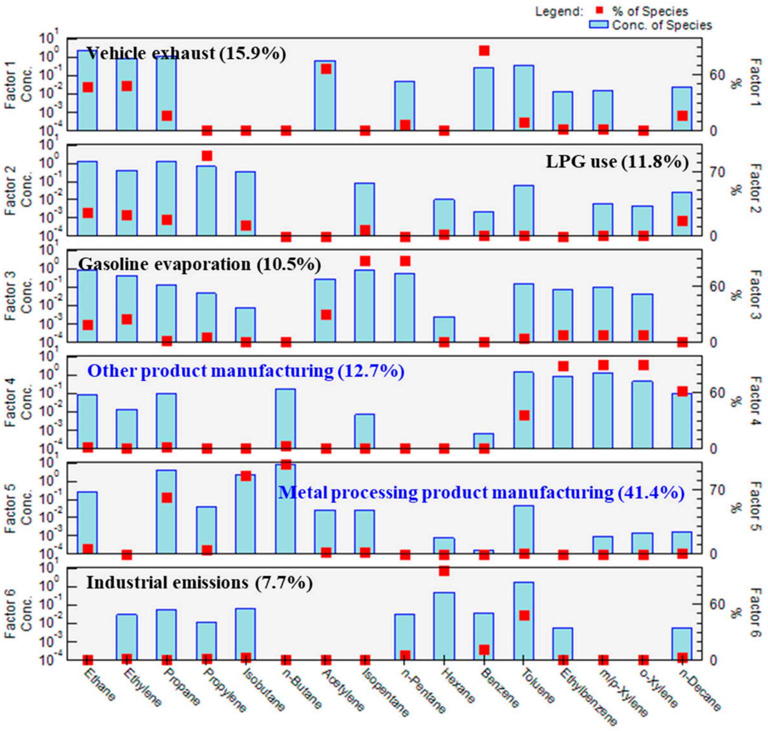

Figure S1. Refined PMF source profiles at the GS based on PRTR emission linkage. Source labels shown in blue indicate PMF factors that were reinterpreted based on linkage with local PRTR emission information, where labels shown in black indicate factors whose original source interpretations were retained.

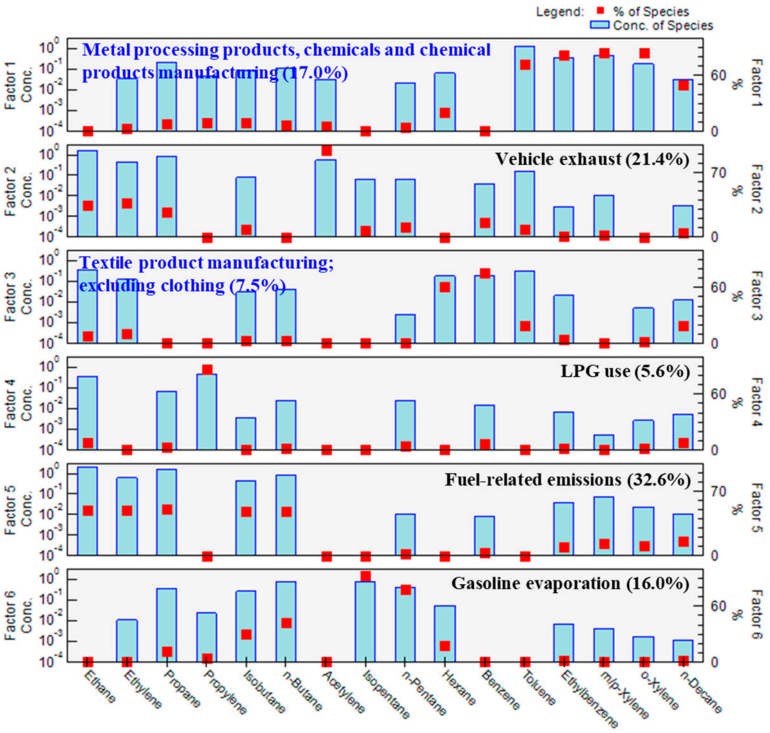

Figure S2. Refined PMF source profiles at the GJ based on PRTR emission linkage. Source labels shown in blue indicate PMF factors that were reinterpreted based on linkage with local PRTR emission information, where labels shown in black indicate factors whose original source interpretations were retained.

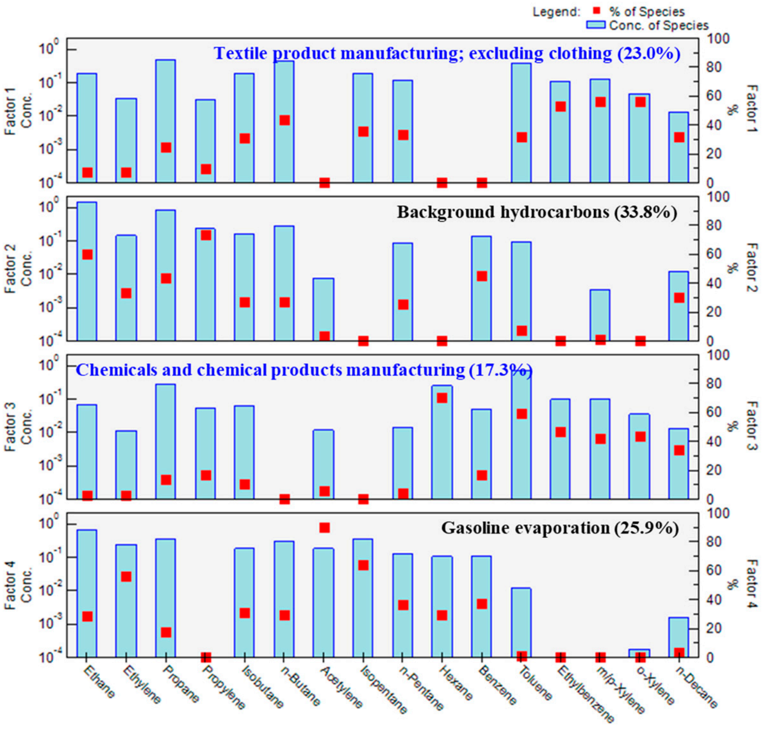

Figure S3. Refined PMF source profiles at the BHS based on PRTR emission linkage. Source labels shown in blue indicate PMF factors that were reinterpreted based on link-

age with local PRTR emission information, where labels shown in black indicate factors whose original source interpretations were retained.

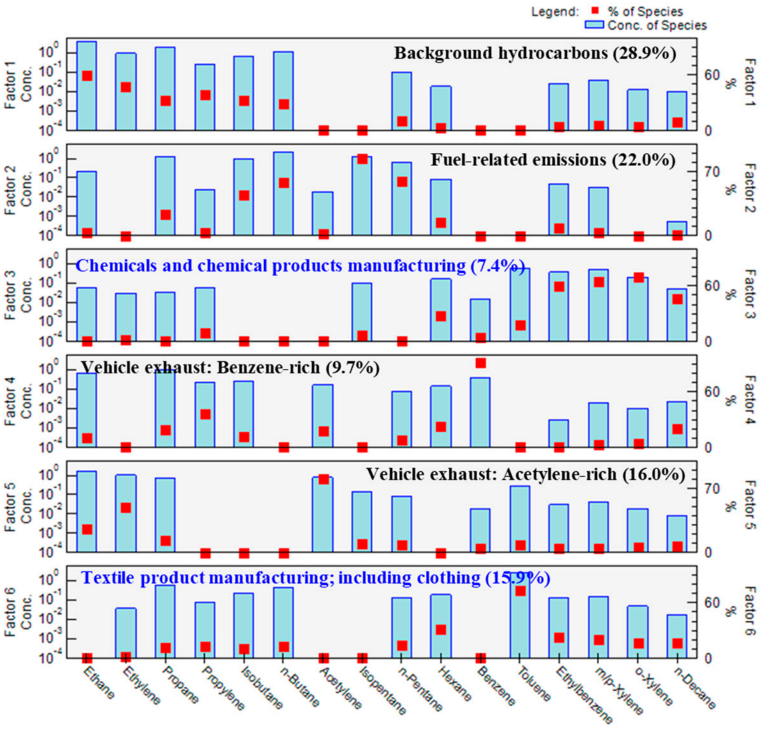

Figure S4. Refined PMF source profiles at the JN based on PRTR emission linkage. Source labels shown in blue indicate PMF factors that were reinterpreted based on linkage with local PRTR emission information, where labels shown in black indicate factors whose original source interpretations were retained.

Table S4. Detection frequency of 56 types of VOCs by measurement sites.

VOCs	Group	Measurement Sites							
		GS		GJ		BHS		JN	
		N	Detection Frequency (%)	N	Detection Frequency (%)	N	Detection Frequency (%)	N	Detection Frequency (%)
Ethane	Alkane	38,416	99.96	43,788	99.99	41,067	99.90	42,572	99.84
Ethylene	Alkene	38,416	99.24	43,794	99.17	41,067	86.91	42,572	98.86
Propane	Alkane	38,416	99.86	43,773	98.57	41,068	99.12	42,572	99.88
Propylene	Alkene	38,416	91.85	43,793	96.85	41,067	90.23	42,572	97.18
Isobutane	Alkane	38,416	99.17	43,776	99.50	41,067	97.66	42,572	99.63
n-Butane	Alkane	38,416	98.08	43,776	97.87	41,067	93.85	42,572	98.83
Acetylene	Alkyne	38,416	93.49	43,794	85.21	41,067	58.47	42,572	91.85
tans-2-Butene	Alkene	38,416	29.55	43,794	28.69	41,068	12.37	42,572	46.25
1-Butene	Alkene	38,416	64.31	43,794	37.51	41,068	48.88	42,572	77.59
cis-2-Butene	Alkene	38,416	38.17	43,794	50.80	41,068	28.41	42,572	43.37
Cyclopentane	Alkane	38,416	81.05	43,794	55.61	41,068	43.23	42,572	79.78
Isopentane	Alkane	38,416	94.83	43,794	93.70	41,068	86.80	42,572	93.93
n-Pentane	Alkane	38,413	96.63	43,793	90.13	41,068	82.15	42,572	98.93
trans-2-Pentene	Alkene	38,415	29.48	43,794	17.43	41,068	2.81	42,572	38.49
1-Pentene	Alkene	38,416	30.63	43,794	9.71	41,068	3.20	42,572	28.21
cis-2-Pentene	Alkene	38,416	12.85	43,794	8.16	41,068	11.07	42,572	15.50
2,2-Dimethylbutane	Alkane	38,416	39.37	43,794	21.03	41,068	8.72	42,572	39.52
2,3-Dimethylbutane	Alkane	38,383	47.30	43,794	31.81	41,067	13.04	42,572	60.91
2-Methylpentane	Alkane	38,385	78.82	43,794	82.21	41,068	45.40	42,572	80.52
3-Methylpentane	Alkane	38,385	62.85	43,794	47.21	41,068	18.39	42,572	64.95
Isoprene	Alkene	38,385	63.38	43,794	47.68	41,068	30.56	42,572	50.83

https://doi.org/10.3390/toxics14090799

1-Hexane	Alkene	38,435	19.95	43,794	26.88	41,068	5.73	42,572	18.61
Hexane	Alkane	39,298	82.03	43,728	64.78	41,094	77.87	42,571	97.26
Methylcyclopentane	Alkane	39,298	59.21	43,728	46.49	41,094	23.93	42,570	74.69
2,4-Dimethylpentane	Alkane	39,298	2.69	43,728	0.30	41,094	2.71	42,570	5.08
Benzene	Aromatic	39,298	78.69	43,727	70.29	41,093	87.27	42,477	91.41
Cyclohexane	Alkane	39,298	35.50	43,728	30.28	41,094	11.47	42,570	36.74
2-Methylhexane	Alkane	39,298	25.34	43,728	23.00	41,094	8.11	42,570	37.96
2,3-Dimethylpentane	Alkane	39,298	4.46	43,728	3.40	41,094	0.96	42,570	12.09
3-Methylhexane	Alkane	39,298	35.01	43,728	31.30	41,094	15.10	42,570	47.21
2,2,4-Trimethylpentane	Alkane	39,298	22.09	43,728	20.60	41,094	5.13	42,570	31.38
n-Heptane	Alkane	39,298	47.32	43,728	39.98	41,094	15.16	42,570	54.89
Methylcyclohexane	Alkane	39,298	47.52	43,728	38.44	41,094	15.61	42,570	52.40
2,3,4-Trimethylpentane	Alkane	39,298	8.88	43,728	14.67	41,094	1.03	42,570	15.04
Toluene	Aromatic	39,298	99.96	43,728	98.63	41,094	96.89	42,570	100.00
2-Methylheptane	Alkane	39,298	13.03	43,728	14.15	41,094	2.22	42,570	7.15
3-Methylheptane	Alkane	39,298	19.30	43,728	10.54	41,094	0.84	42,570	6.64
n-Octane	Alkane	39,298	43.06	43,728	39.84	41,094	15.05	42,570	23.68
Ethylbenzene	Aromatic	39,042	98.44	43,728	96.10	41,094	81.08	42,570	96.08
m,p-Xylene	Aromatic	39,042	98.86	43,728	93.49	41,094	71.08	42,570	96.20
Styrene	Aromatic	39,298	53.92	43,728	40.14	41,094	4.32	42,570	11.76
o-Xylene	Aromatic	39,042	95.93	43,728	85.15	41,094	60.02	42,570	89.82
n-Nonane	Alkane	39,298	51.28	43,728	54.95	41,094	16.96	42,570	34.91
Isopropylbenzene	Aromatic	39,298	12.04	43,728	1.25	41,094	0.50	42,570	2.92
n-Propylbenzene	Aromatic	39,298	14.86	43,728	5.12	41,094	4.28	42,570	2.36
m-Ethyltoluene	Aromatic	39,298	63.04	43,728	40.92	41,094	16.56	42,570	20.18
p-Ethyltoluene	Aromatic	39,042	19.30	43,728	15.34	41,094	0.95	42,570	7.55

https://doi.org/10.3390/toxics14090799

1,3,5-Trimethylbenzene	Aromatic	39,298	35.83	43,728	24.22	41,094	4.11	42,570	6.27
o-Ethyltoluene	Aromatic	39,042	23.03	43,728	19.58	41,094	0.95	42,570	6.14
1,2,4-Trimethylbenzene	Aromatic	39,298	86.34	43,728	41.58	41,094	29.21	42,570	34.30
n-Decane	Alkane	39,298	92.14	43,728	51.85	41,094	59.09	42,570	62.52
1,2,3-Trimethylbenzene	Aromatic	39,298	67.14	43,728	41.57	41,094	38.72	42,570	17.52
m-Diethylbenzene	Aromatic	39,298	18.22	43,728	1.55	41,094	0.79	42,570	8.94
p-Diethylbenzene	Aromatic	39,298	25.71	43,728	2.62	41,094	1.02	42,570	2.92
n-Undecane	Alkane	39,298	68.54	43,728	27.76	41,094	23.49	42,570	9.62
n-Dodecane	Alkane	39,298	74.52	43,090	27.47	41,094	7.24	42,570	7.64

https://doi.org/10.3390/toxics14090799

4. Sensitivity Analysis for the VOC Selection Criterion

4.1. Sensitivity Analysis Method

To evaluate the potential selection bias associated with the 50% detection frequency criterion used for VOC selection, an additional sensitivity analysis was conducted. In the primary analysis, 16 VOC species with detection frequencies greater than 50% were selected from the 56 VOC species measured at the four PAMS sites to ensure the reliability and comparability of the long-term dataset. However, the application of this criterion may exclude compounds with relatively low detection frequencies but potentially relevant toxicological effects, thereby leading to an underestimation of cumulative health risks. Therefore, an MDL-based conservative sensitivity analysis was performed to quantify the pontential influence of the excluded VOC species on the final total excess cancer risk (TECR) and hazard index (HI).

Among the VOC species excluded by the 50% detection frequency criterion, compounds for which applicable inhalation toxicity reference values were available were identified based on the same toxicity-data framework used in the primary health risk assessment. For the sensitivity analysis, measured concentrations at or above the corresponding site-specific MDLs were retained as observed, whereas concentrations below the MDL were conservatively replaced with the corresponding site-specific MDL values. Missing observations for which no measurement was available were not substituted and were excluded from the calculation. The resulting concentration distributions were used to calculate the 50th percentile concentration for the central tendency exposure (CTE) scenario and the 95th percentile concentration for the reasonable maximum exposure (RME) scenario, consistent with the exposure assessment approach used in the primary analysis.

The excess cancer risk (ECR) and hazard quotient (HQ) for the additionally considered VOC species were subsequently calculated using the same dose–response relationships, exposure factors, and risk characterization procedures as those used in the primary health risk assessment. The calculated ECR and HQ values of the excluded VOCs were added to the corresponding baseline TECR and HI values for each measurement site, age group, and exposure scenario. The resulting sensitivity estimates were then compared with the baseline estimates to determine the percentage change in TECR and HI and to evaluate whether the inclusion of the excluded VOCs altered the overall health risk interpretation. The VOC species and toxicity reference values included in the sensitivity analysis are summarized in Table S4.

Table S5. Toxicity information of VOC species included in the MDL-based sensitivity analysis.

VOCs	IUR ((°g/m^3)$^{-1}$)	RfC (mg/m^3)	Reference
1,2,3-Trimethylbenzene	-	0.06	IRIS
1,2,4-Trimethylbenzene	-	0.06	IRIS
1,3,5-Trimethylbenzene	-	0.06	IRIS
Cyclohexane	-	6	IRIS
Isopropylbenzene	-	0.4	IRIS
Styrene	-	1	IRIS
n-Heptane	-	0.4	PPRTV
n-Nonane	-	0.02	PPRTV
Methylcyclohexane	-	0.1	PPRTV
n-Propylbenzene	-	1	PPRTV

4.2. Sensitivity Analysis Results

Among the VOC species excluded by the 50% detection frequency criterion, 10 compounds had applicable inhalation toxicity reference values and were therefore included in the MDL-based sensitivity analysis (Table S4). Under the toxicity-data framework applied in this study, these compounds had reference concentrations (RfCs) applicable to the assessment of non-carcinogenic effects, whereas no applicable quantitative inhalation unit risk (IUR) values were identified for the

https://doi.org/10.3390/toxics14090799

assessment of carcinogenic effects. Consequently, the inclusion of these compounds did not result in an additional ECR, and the sensitivity TECR estimates were identical to the corresponding baseline TECR estimates across all measurement sites, age groups, and exposure scenarios.

In contrast, the inclusion of the excluded VOC species increased the estimated HI across all measurement sites and exposure scenarios. Compared with the baseline assessment, the HI increased by 36.12~77.58% under the conservative MDL-based sensitivity scenario. The magnitude of the increase varied among measurement sites, ranging from 42.92~77.58% at GS, 39.88~46.30% at GJ, 36.12~44.93% at BHS, and 43.41~54.86% at JN. Despite these increases, all sensitivity HI estimates remained below the acceptable criterion of 1. The highest sensitivity HI was 0.184 at the GS site, indicating that the inclusion of the excluded VOC species did not alter the interpretation of non-carcinogenic health risks.

Overall, the sensitivity analysis indicates that application of the 50% detection frequency criterion may result in some underestimation of cumulative non-carcinogenic health risks. However, even under the conservative assumption that concentrations below the MDL were present at the corresponding MDL values, the overall health risk classification remianed unchanged. Similarly, the TECR classification was unaffected because no additional quantitative inhalation cancer risk could be estimated for the excluded VOC species under the toxicity-data framework applied in this study. These findings suggest that although the VOC selection criterion influences the magnitude of the estimated HI, it does not materially affect the principal health risk interpretation of the present study.

https://doi.org/10.3390/toxics14090799

Table S6. Comparison of baseline and MDL-based sensitivity estimates of TECR and HI by age group at the GS site.

Age group	Baseline-TECR		Sensitivity-TECR		Change (%)		Baseline-HI		Sensitivity-HI		Change (%)	
	CTE[a]	RME[b]	CTE	RME	CTE	RME	CTE	RME	CTE	RME	CTE	RME
Infant	2.2(f)10^{-8}	3.96(f)10^{-7}	2.23(f)10^{-8}	3.96(f)10^{-7}	0.00	0.00	6.69(f)10^{-3}	1.04(f)10^{-1}	9.56(f)10^{-3}	1.84(f)10^{-1}	42.97	77.24
Young children	1.81(f)10^{-8}	3.64(f)10^{-7}	1.81(f)10^{-8}	3.64(f)10^{-7}	0.00	0.00	4.06(f)10^{-3}	7.14(f)10^{-2}	5.81(f)10^{-3}	1.27(f)10^{-1}	43.00	77.54
Elementary school children	1.64(f)10^{-8}	2.36(f)10^{-7}	1.64(f)10^{-8}	2.36(f)10^{-7}	0.00	0.00	2.46(f)10^{-3}	3.09(f)10^{-2}	3.52(f)10^{-3}	5.49(f)10^{-2}	42.96	77.58
Adolescent	1.09(f)10^{-8}	1.50(f)10^{-7}	1.09(f)10^{-8}	1.50(f)10^{-7}	0.00	0.00	1.63(f)10^{-3}	1.96(f)10^{-2}	2.33(f)10^{-3}	3.48(f)10^{-2}	42.92	77.53
Adults	2.52(f)10^{-7}	5.21(f)10^{-6}	2.52(f)10^{-7}	5.21(f)10^{-6}	0.00	0.00	4.93(f)10^{-3}	8.90(f)10^{-2}	7.05(f)10^{-3}	1.58(f)10^{-1}	43.02	77.48
Elderly	2.67(f)10^{-7}	2.45(f)10^{-6}	2.67(f)10^{-7}	2.45(f)10^{-6}	0.00	0.00	1.20(f)10^{-2}	9.61(f)10^{-2}	1.72(f)10^{-2}	1.71(f)10^{-1}	43.00	77.52

[a]CTE: central tendency exposure; [b]RME: reasonable maximum exposure.

Table S7. Comparison of baseline and MDL-based sensitivity estimates of TECR and HI by age group at the GJ site.

Age group	Baseline-TECR		Sensitivity-TECR		Change (%)		Baseline-HI		Sensitivity-HI		Change (%)	
	CTE[a]	RME[b]	CTE	RME	CTE	RME	CTE	RME	CTE	RME	CTE	RME
Infant	1.24(f)10^{-8}	1.69(f)10^{-7}	1.24(f)10^{-8}	1.69(f)10^{-7}	0.00	0.00	3.57(f)10^{-3}	4.99(f)10^{-2}	5.22(f)10^{-3}	6.98(f)10^{-2}	46.21	39.94
Young children	1.00(f)10^{-8}	1.56(f)10^{-7}	1.00(f)10^{-8}	1.56(f)10^{-7}	0.00	0.00	2.17(f)10^{-3}	3.44(f)10^{-2}	3.17(f)10^{-3}	4.81(f)10^{-2}	46.18	39.92
Elementary school children	9.09(f)10^{-9}	1.01(f)10^{-7}	9.09(f)10^{-9}	1.01(f)10^{-7}	0.00	0.00	1.31(f)10^{-3}	1.49(f)10^{-2}	1.92(f)10^{-3}	2.08(f)10^{-2}	46.30	39.92
Adolescent	6.02(f)10^{-9}	6.41(f)10^{-8}	6.02(f)10^{-9}	6.41(f)10^{-8}	0.00	0.00	8.68(f)10^{-4}	9.45(f)10^{-3}	1.27(f)10^{-3}	1.32(f)10^{-2}	46.26	39.89
Adults	1.40(f)10^{-7}	2.23(f)10^{-6}	1.40(f)10^{-7}	2.23(f)10^{-6}	0.00	0.00	2.63(f)10^{-3}	4.29(f)10^{-2}	3.85(f)10^{-3}	6.00(f)10^{-2}	46.29	39.88
Elderly	1.48(f)10^{-7}	1.05(f)10^{-6}	1.48(f)10^{-7}	1.05(f)10^{-6}	0.00	0.00	6.41(f)10^{-3}	4.63(f)10^{-2}	9.37(f)10^{-3}	6.48(f)10^{-2}	46.20	39.92

https://doi.org/10.3390/toxics14090799

Table S8. Comparison of baseline and MDL-based sensitivity estimates of TECR and HI by age group at the BHS site.

Age group	Baseline-TECR		Sensitivity-TECR		Change (%)		Baseline-HI		Sensitivity-HI		Change (%)	
	CTE[a]	RME[b]	CTE	RME	CTE	RME	CTE	RME	CTE	RME	CTE	RME
Infant	$8.36(f)10^{-9}$	$1.08(f)10^{-7}$	$8.36(f)10^{-9}$	$1.08(f)10^{-7}$	0.00	0.00	$2.87(f)10^{-3}$	$3.27(f)10^{-2}$	$4.16(f)10^{-3}$	$4.45(f)10^{-2}$	44.93	36.12
Young children	$6.77(f)10^{-9}$	$9.96(f)10^{-8}$	$6.77(f)10^{-9}$	$9.96(f)10^{-8}$	0.00	0.00	$1.75(f)10^{-3}$	$2.25(f)10^{-2}$	$2.53(f)10^{-3}$	$3.06(f)10^{-2}$	44.76	36.18
Elementary school children	$6.15(f)10^{-9}$	$6.47(f)10^{-8}$	$6.15(f)10^{-9}$	$6.47(f)10^{-8}$	0.00	0.00	$1.06(f)10^{-3}$	$9.75(f)10^{-3}$	$1.53(f)10^{-3}$	$1.33(f)10^{-2}$	44.73	36.16
Adolescent	$4.07(f)10^{-9}$	$4.10(f)10^{-8}$	$4.07(f)10^{-9}$	$4.10(f)10^{-8}$	0.00	0.00	$6.99(f)10^{-4}$	$6.18(f)10^{-3}$	$1.01(f)10^{-3}$	$8.41(f)10^{-3}$	44.90	36.16
Adults	$9.46(f)10^{-8}$	$1.43(f)10^{-6}$	$9.46(f)10^{-8}$	$1.43(f)10^{-6}$	0.00	0.00	$2.12(f)10^{-3}$	$2.80(f)10^{-2}$	$3.07(f)10^{-3}$	$3.81(f)10^{-2}$	44.88	36.21
Elderly	$1.00(f)10^{-7}$	$6.70(f)10^{-7}$	$1.00(f)10^{-7}$	$6.70(f)10^{-7}$	0.00	0.00	$5.16(f)10^{-3}$	$3.03(f)10^{-2}$	$7.47(f)10^{-3}$	$4.13(f)10^{-2}$	44.86	36.15

Table S9. Comparison of baseline and MDL-based sensitivity estimates of TECR and HI by age group at the JN site.

Age group	Baseline-TECR		Sensitivity-TECR		Change (%)		Baseline-HI		Sensitivity-HI		Change (%)	
	CTE[a]	RME[b]	CTE	RME	CTE	RME	CTE	RME	CTE	RME	CTE	RME
Infant	$2.13(f)10^{-8}$	$2.26(f)10^{-7}$	$2.13(f)10^{-8}$	$2.26(f)10^{-7}$	0.00	0.00	$6.43(f)10^{-3}$	$6.56(f)10^{-2}$	$9.94(f)10^{-3}$	$9.41(f)10^{-2}$	54.66	43.46
Young children	$1.73(f)10^{-8}$	$2.08(f)10^{-7}$	$1.73(f)10^{-8}$	$2.08(f)10^{-7}$	0.00	0.00	$3.90(f)10^{-3}$	$4.52(f)10^{-2}$	$6.03(f)10^{-3}$	$6.48(f)10^{-2}$	54.74	43.47
Elementary school children	$1.57(f)10^{-8}$	$1.35(f)10^{-7}$	$1.57(f)10^{-8}$	$1.35(f)10^{-7}$	0.00	0.00	$2.36(f)10^{-3}$	$1.96(f)10^{-2}$	$3.65(f)10^{-3}$	$2.81(f)10^{-2}$	54.76	43.41
Adolescent	$1.04(f)10^{-8}$	$8.57(f)10^{-8}$	$1.04(f)10^{-8}$	$8.57(f)10^{-8}$	0.00	0.00	$1.56(f)10^{-3}$	$1.24(f)10^{-2}$	$2.42(f)10^{-3}$	$1.78(f)10^{-2}$	54.84	43.49
Adults	$2.41(f)10^{-7}$	$2.98(f)10^{-6}$	$2.41(f)10^{-7}$	$2.98(f)10^{-6}$	0.00	0.00	$4.74(f)10^{-3}$	$5.63(f)10^{-2}$	$7.33(f)10^{-3}$	$8.08(f)10^{-2}$	54.71	43.47
Elderly	$2.55(f)10^{-7}$	$1.40(f)10^{-6}$	$2.55(f)10^{-7}$	$1.40(f)10^{-6}$	0.00	0.00	$1.15(f)10^{-2}$	$6.08(f)10^{-2}$	$1.78(f)10^{-2}$	$8.72(f)10^{-2}$	54.86	43.48

Table S10. Results of ECR for individual VOCs at each measurement site (infant).

VOCs	Measurement Site							
	GS		GJ		BHS		JN	
	CTE[a]	RME[b]	CTE	RME	CTE	RME	CTE	RME
Benzene	5.57×10^{-9}	5.90×10^{-8}	3.60×10^{-9}	4.85×10^{-8}	4.92×10^{-9}	4.36×10^{-8}	6.89×10^{-9}	5.80×10^{-8}
Ethylbenzene	1.67×10^{-8}	3.37×10^{-7}	8.76×10^{-9}	1.21×10^{-7}	3.44×10^{-9}	6.48×10^{-8}	1.44×10^{-8}	1.68×10^{-7}
TECR	2.23×10^{-8}	3.96×10^{-7}	1.24×10^{-8}	1.69×10^{-7}	8.36×10^{-9}	1.08×10^{-7}	2.13×10^{-8}	2.26×10^{-7}

Table S11. Results of ECR for Individual VOCs at each measurement site (young children).

VOCs	Measurement Site							
	GS		GJ		BHS		JN	
	CTE	RME	CTE	RME	CTE	RME	CTE	RME
Benzene	4.51×10^{-9}	5.42×10^{-8}	2.92×10^{-9}	4.46×10^{-8}	3.99×10^{-9}	4.01×10^{-8}	5.07×10^{-9}	3.46×10^{-8}
Ethylbenzene	1.35×10^{-8}	3.10×10^{-7}	7.09×10^{-9}	1.11×10^{-7}	2.78×10^{-9}	5.95×10^{-8}	1.06×10^{-8}	1.01×10^{-7}
TECR	1.81×10^{-8}	3.64×10^{-7}	1.00×10^{-8}	1.56×10^{-7}	6.77×10^{-9}	9.96×10^{-8}	1.57×10^{-8}	1.35×10^{-7}

Table S12. Results of ECR for individual VOCs at each measurement site (elementary school children).

VOCs	Measurement Site							
	GS		GJ		BHS		JN	
	CTE	RME	CTE	RME	CTE	RME	CTE	RME
Benzene	4.10×10^{-9}	3.52×10^{-8}	2.65×10^{-9}	2.90×10^{-8}	3.62×10^{-9}	2.60×10^{-8}	5.07×10^{-9}	3.46×10^{-8}
Ethylbenzene	1.23×10^{-8}	2.01×10^{-7}	6.44×10^{-9}	7.22×10^{-8}	2.53×10^{-9}	3.87×10^{-8}	1.06×10^{-8}	1.01×10^{-7}
TECR	1.64×10^{-8}	2.36×10^{-7}	9.09×10^{-9}	1.01×10^{-7}	6.15×10^{-9}	6.47×10^{-8}	1.57×10^{-8}	1.35×10^{-7}

https://doi.org/10.3390/toxics14090799

Table S13. Results of ECR for individual VOCs at each measurement site (adolescent).

VOCs	Measurement Site							
	GS		GJ		BHS		JN	
	CTE	RME	CTE	RME	CTE	RME	CTE	RME
Benzene	2.71×10^{-9}	2.23×10^{-8}	1.75×10^{-9}	1.84×10^{-8}	2.40×10^{-9}	1.65×10^{-8}	3.35×10^{-9}	2.19×10^{-8}
Ethylbenzene	8.14×10^{-9}	1.28×10^{-7}	4.26×10^{-9}	4.58×10^{-8}	1.67×10^{-9}	2.45×10^{-8}	7.03×10^{-9}	6.38×10^{-8}
TECR	1.09×10^{-8}	1.50×10^{-7}	6.02×10^{-9}	6.41×10^{-8}	4.07×10^{-9}	4.10×10^{-8}	1.04×10^{-8}	8.57×10^{-8}

Table S14. Results of ECR for individual VOCs at each measurement site (adults).

VOCs	Measurement Site							
	GS		GJ		BHS		JN	
	CTE	RME	CTE	RME	CTE	RME	CTE	RME
Benzene	6.31×10^{-8}	7.77×10^{-7}	4.08×10^{-8}	6.39×10^{-7}	5.57×10^{-8}	5.74×10^{-7}	7.80×10^{-8}	7.63×10^{-7}
Ethylbenzene	7.77×10^{-7}	4.44×10^{-6}	9.91×10^{-8}	1.59×10^{-6}	3.89×10^{-8}	8.52×10^{-7}	1.63×10^{-7}	2.22×10^{-6}
TECR	2.52×10^{-7}	5.21×10^{-6}	1.40×10^{-7}	2.23×10^{-6}	9.46×10^{-8}	1.43×10^{-6}	2.41×10^{-7}	2.98×10^{-6}

Table S15. Results of ECR for Individual VOCs at each measurement site (elderly).

VOCs	Measurement Site							
	GS		GJ		BHS		JN	
	CTE	RME	CTE	RME	CTE	RME	CTE	RME
Benzene	6.67×10^{-8}	3.65×10^{-7}	4.31×10^{-8}	3.00×10^{-7}	5.89×10^{-8}	2.70×10^{-7}	8.25×10^{-8}	3.59×10^{-7}
Ethylbenzene	2.00×10^{-7}	2.08×10^{-6}	1.05×10^{-7}	7.48×10^{-7}	4.11×10^{-8}	4.00×10^{-7}	1.73×10^{-7}	1.04×10^{-6}
TECR	2.67×10^{-7}	2.45×10^{-6}	1.48×10^{-7}	1.05×10^{-6}	1.00×10^{-7}	6.70×10^{-7}	2.55×10^{-7}	1.40×10^{-6}

https://doi.org/10.3390/toxics14090799

Table S16. Results of HQ for individual VOCs at each measurement site (infant).

VOCs	Measurement Site							
	GS		GJ		BHS		JN	
	CTE	RME	CTE	RME	CTE	RME	CTE	RME
Propylene	2.70×10^{-5}	2.79×10^{-4}	1.95×10^{-5}	1.54×10^{-4}	1.35×10^{-5}	1.54×10^{-4}	2.55×10^{-5}	2.36×10^{-4}
n-Pentane	1.16×10^{-4}	1.50×10^{-3}	8.33×10^{-5}	1.20×10^{-3}	5.09×10^{-5}	7.14×10^{-4}	1.81×10^{-4}	2.08×10^{-3}
Hexane	1.19×10^{-4}	2.28×10^{-3}	5.93×10^{-5}	1.75×10^{-3}	8.57×10^{-5}	1.40×10^{-3}	2.04×10^{-4}	2.37×10^{-3}
Benzene	2.36×10^{-3}	2.50×10^{-2}	1.53×10^{-3}	2.06×10^{-2}	2.09×10^{-3}	1.85×10^{-2}	2.92×10^{-3}	2.46×10^{-2}
Toluene	1.69×10^{-4}	2.50×10^{-3}	7.27×10^{-5}	1.23×10^{-3}	4.14×10^{-5}	7.70×10^{-4}	1.41×10^{-4}	2.10×10^{-3}
Ethylbenzene	1.87×10^{-4}	3.77×10^{-3}	9.81×10^{-5}	1.35×10^{-3}	3.85×10^{-5}	7.25×10^{-4}	1.62×10^{-4}	1.89×10^{-3}
m/p-Xylene	2.68×10^{-3}	4.94×10^{-2}	1.24×10^{-3}	1.68×10^{-2}	4.26×10^{-4}	7.80×10^{-3}	2.07×10^{-3}	2.32×10^{-2}
o-Xylene	1.02×10^{-3}	1.89×10^{-2}	4.70×10^{-4}	6.84×10^{-3}	1.29×10^{-4}	2.60×10^{-3}	7.26×10^{-4}	9.03×10^{-3}
HI	6.69×10^{-3}	1.04×10^{-1}	3.57×10^{-3}	4.99×10^{-2}	2.87×10^{-3}	3.27×10^{-2}	6.43×10^{-3}	6.56×10^{-2}

Table S17. Results of HQ for individual VOCs at each measurement site (young children).

VOCs	Measurement Site							
	GS		GJ		BHS		JN	
	CTE	RME	CTE	RME	CTE	RME	CTE	RME
Propylene	1.64×10^{-5}	1.93×10^{-4}	1.18×10^{-5}	1.06×10^{-4}	8.20×10^{-6}	1.06×10^{-4}	1.55×10^{-5}	1.63×10^{-4}
n-Pentane	7.03×10^{-5}	1.04×10^{-3}	5.06×10^{-5}	8.30×10^{-4}	3.09×10^{-5}	4.92×10^{-4}	1.10×10^{-4}	1.43×10^{-3}
Hexane	7.20×10^{-5}	1.57×10^{-3}	3.60×10^{-5}	1.21×10^{-3}	5.21×10^{-5}	9.62×10^{-4}	1.24×10^{-4}	1.63×10^{-3}
Benzene	1.44×10^{-3}	1.73×10^{-2}	9.29×10^{-4}	1.42×10^{-2}	1.27×10^{-3}	1.28×10^{-2}	1.78×10^{-3}	1.70×10^{-2}
Toluene	1.03×10^{-4}	1.72×10^{-3}	4.41×10^{-5}	8.49×10^{-4}	2.51×10^{-5}	5.31×10^{-4}	8.57×10^{-5}	1.45×10^{-3}
Ethylbenzene	1.14×10^{-4}	2.60×10^{-3}	5.96×10^{-5}	9.33×10^{-4}	2.34×10^{-5}	5.00×10^{-4}	9.83×10^{-5}	1.30×10^{-3}
m/p-Xylene	1.63×10^{-3}	3.40×10^{-2}	7.51×10^{-4}	1.16×10^{-2}	2.59×10^{-4}	5.38×10^{-3}	1.25×10^{-3}	1.60×10^{-2}
o-Xylene	6.21×10^{-4}	1.30×10^{-2}	2.86×10^{-4}	4.71×10^{-3}	7.86×10^{-5}	1.79×10^{-3}	4.41×10^{-4}	6.22×10^{-3}

https://doi.org/10.3390/toxics14090799

HI	4.06×10^{-3}	7.14×10^{-2}	2.17×10^{-3}	3.44×10^{-2}	1.75×10^{-3}	2.25×10^{-2}	3.90×10^{-3}	4.52×10^{-2}

Table S18. Results of HQ for individual VOCs at each measurement site (elementary school children).

VOCs	Measurement Site							
	GS		GJ		BHS		JN	
	CTE	RME	CTE	RME	CTE	RME	CTE	RME
Propylene	9.93×10^{-6}	8.34×10^{-5}	7.17×10^{-6}	4.60×10^{-5}	4.96×10^{-6}	4.60×10^{-5}	9.38×10^{-6}	7.04×10^{-5}
n-Pentane	4.26×10^{-5}	4.48×10^{-4}	3.06×10^{-5}	3.59×10^{-4}	1.87×10^{-5}	2.13×10^{-4}	6.64×10^{-5}	6.21×10^{-4}
Hexane	4.36×10^{-5}	6.82×10^{-4}	2.18×10^{-5}	5.24×10^{-4}	3.15×10^{-5}	4.17×10^{-4}	7.51×10^{-5}	7.07×10^{-4}
Benzene	8.69×10^{-4}	7.47×10^{-3}	5.62×10^{-4}	6.14×10^{-3}	7.68×10^{-4}	5.52×10^{-3}	1.08×10^{-3}	7.34×10^{-3}
Toluene	6.21×10^{-5}	7.46×10^{-4}	2.67×10^{-5}	3.68×10^{-4}	1.52×10^{-5}	2.30×10^{-4}	5.19×10^{-5}	6.27×10^{-4}
Ethylbenzene	6.89×10^{-5}	1.13×10^{-3}	3.61×10^{-5}	4.04×10^{-4}	1.42×10^{-5}	2.16×10^{-4}	5.95×10^{-5}	5.63×10^{-4}
m/p-Xylene	9.87×10^{-4}	1.47×10^{-2}	4.55×10^{-4}	5.02×10^{-3}	1.57×10^{-4}	2.33×10^{-3}	7.60×10^{-4}	6.94×10^{-3}
o-Xylene	3.76×10^{-4}	5.63×10^{-3}	1.73×10^{-4}	2.04×10^{-3}	4.76×10^{-5}	7.77×10^{-4}	2.67×10^{-4}	2.69×10^{-3}
HI	2.46×10^{-3}	3.09×10^{-2}	1.31×10^{-3}	1.49×10^{-2}	1.06×10^{-3}	9.75×10^{-3}	2.36×10^{-3}	1.96×10^{-2}

Table S19. Results of HQ for individual VOCs at each measurement site (adolescent).

VOCs	Measurement Site							
	GS		GJ		BHS		JN	
	CTE	RME	CTE	RME	CTE	RME	CTE	RME
Propylene	6.57×10^{-6}	5.29×10^{-5}	4.74×10^{-6}	2.92×10^{-5}	3.29×10^{-6}	2.92×10^{-5}	6.21×10^{-6}	4.47×10^{-5}
n-Pentane	2.82×10^{-5}	2.84×10^{-4}	2.03×10^{-5}	2.28×10^{-4}	1.24×10^{-5}	1.35×10^{-4}	4.39×10^{-5}	3.94×10^{-4}
Hexane	2.88×10^{-5}	4.32×10^{-4}	1.44×10^{-5}	3.32×10^{-4}	2.09×10^{-5}	2.64×10^{-4}	4.97×10^{-5}	4.48×10^{-4}
Benzene	5.75×10^{-4}	4.74×10^{-3}	3.72×10^{-4}	3.89×10^{-3}	5.08×10^{-4}	3.50×10^{-3}	7.12×10^{-4}	4.65×10^{-3}
Toluene	4.11×10^{-5}	4.73×10^{-4}	1.77×10^{-5}	2.33×10^{-4}	1.01×10^{-5}	1.46×10^{-4}	3.43×10^{-5}	3.98×10^{-4}
Ethylbenzene	4.56×10^{-5}	7.14×10^{-4}	2.39×10^{-5}	2.56×10^{-4}	9.37×10^{-6}	1.37×10^{-4}	3.94×10^{-5}	3.57×10^{-4}

m/p-Xylene	6.53×10^{-4}	9.34×10^{-3}	3.01×10^{-4}	3.18×10^{-3}	1.04×10^{-4}	1.48×10^{-3}	5.03×10^{-4}	4.40×10^{-3}
o-Xylene	2.49×10^{-4}	3.57×10^{-3}	1.14×10^{-4}	1.29×10^{-3}	3.15×10^{-5}	4.93×10^{-4}	1.77×10^{-4}	1.71×10^{-3}
HI	1.63×10^{-3}	1.96×10^{-2}	8.68×10^{-4}	9.45×10^{-3}	6.99×10^{-4}	6.18×10^{-3}	1.56×10^{-3}	1.24×10^{-2}

Table S20. Results of HQ for individual VOCs at each measurement site (adults).

VOCs	Measurement Site							
	GS		GJ		BHS		JN	
	CTE	RME	CTE	RME	CTE	RME	CTE	RME
Propylene	1.99×10^{-5}	2.40×10^{-4}	1.44×10^{-5}	1.32×10^{-4}	9.96×10^{-6}	1.32×10^{-4}	1.88×10^{-5}	2.03×10^{-4}
n-Pentane	8.54×10^{-5}	1.29×10^{-3}	6.15×10^{-5}	1.03×10^{-3}	3.76×10^{-5}	6.13×10^{-4}	1.33×10^{-4}	1.79×10^{-3}
Hexane	8.74×10^{-5}	1.96×10^{-3}	4.37×10^{-5}	1.51×10^{-3}	6.33×10^{-5}	1.20×10^{-3}	1.51×10^{-4}	2.03×10^{-3}
Benzene	1.74×10^{-3}	2.15×10^{-2}	1.13×10^{-3}	1.77×10^{-2}	1.54×10^{-3}	1.59×10^{-2}	2.16×10^{-3}	2.11×10^{-2}
Toluene	1.25×10^{-4}	2.15×10^{-3}	5.36×10^{-5}	1.06×10^{-3}	3.05×10^{-5}	6.61×10^{-4}	1.04×10^{-4}	1.80×10^{-3}
Ethylbenzene	1.38×10^{-4}	3.24×10^{-3}	7.24×10^{-5}	1.16×10^{-3}	2.84×10^{-5}	6.23×10^{-4}	1.19×10^{-4}	1.62×10^{-3}
m/p-Xylene	1.98×10^{-3}	4.24×10^{-2}	9.12×10^{-4}	1.44×10^{-2}	3.14×10^{-4}	6.70×10^{-3}	1.52×10^{-3}	2.00×10^{-2}
o-Xylene	7.54×10^{-4}	1.62×10^{-2}	3.47×10^{-4}	5.87×10^{-3}	9.55×10^{-5}	2.24×10^{-3}	5.35×10^{-4}	7.75×10^{-3}
HI	4.93×10^{-3}	8.90×10^{-2}	2.63×10^{-3}	4.29×10^{-2}	2.12×10^{-3}	2.80×10^{-2}	4.74×10^{-3}	5.63×10^{-2}

https://doi.org/10.3390/toxics14090799

Table S21. Results of HQ for individual VOCs at each measurement site (elderly).

VOCs	Measurement Site							
	GS		GJ		BHS		JN	
	CTE	RME	CTE	RME	CTE	RME	CTE	RME
Propylene	4.85×10^{-5}	2.59×10^{-4}	3.50×10^{-5}	1.43×10^{-4}	2.42×10^{-5}	1.43×10^{-4}	4.58×10^{-5}	2.19×10^{-4}
n-Pentane	2.08×10^{-4}	1.39×10^{-3}	1.50×10^{-4}	1.12×10^{-3}	9.14×10^{-5}	6.62×10^{-4}	3.24×10^{-4}	1.93×10^{-3}
Hexane	2.13×10^{-4}	2.12×10^{-3}	1.06×10^{-4}	1.63×10^{-3}	1.54×10^{-4}	1.29×10^{-3}	3.67×10^{-4}	2.20×10^{-3}
Benzene	4.24×10^{-3}	2.32×10^{-2}	2.74×10^{-3}	1.91×10^{-2}	3.75×10^{-3}	1.72×10^{-2}	5.25×10^{-3}	2.28×10^{-2}
Toluene	3.03×10^{-4}	2.32×10^{-3}	1.30×10^{-4}	1.14×10^{-3}	7.43×10^{-5}	7.15×10^{-4}	2.53×10^{-4}	1.95×10^{-3}
Ethylbenzene	3.36×10^{-4}	3.50×10^{-3}	1.76×10^{-4}	1.26×10^{-3}	6.91×10^{-5}	6.73×10^{-4}	2.90×10^{-4}	1.75×10^{-3}
m/p-Xylene	4.82×10^{-3}	4.58×10^{-2}	2.22×10^{-3}	1.56×10^{-2}	7.64×10^{-4}	7.23×10^{-3}	3.71×10^{-3}	2.16×10^{-2}
o-Xylene	1.83×10^{-3}	1.75×10^{-2}	8.44×10^{-4}	6.34×10^{-3}	2.32×10^{-4}	2.41×10^{-3}	1.30×10^{-3}	8.37×10^{-3}
HI	1.20×10^{-2}	9.61×10^{-2}	6.41×10^{-3}	4.63×10^{-2}	5.16×10^{-3}	3.03×10^{-2}	1.15×10^{-2}	6.08×10^{-2}

https://doi.org/10.3390/toxics14090799

Table S22. Age-specific source-oriented health risk rankings at the GS site.

Age Group	Highest TECR-Contributing Source	Highest HI-Contributing Source	Consistency with Adult Ranking
Infant	Other product manufacturing	Other product manufacturing	Yes
Young children	Other product manufacturing	Other product manufacturing	Yes
Elementary school children	Other product manufacturing	Other product manufacturing	Yes
Adolescent	Other product manufacturing	Other product manufacturing	Yes
Adults	Other product manufacturing	Other product manufacturing	Reference
Elderly	Other product manufacturing	Other product manufacturing	Yes

Table S23. Age-specific source-oriented health risk rankings at the GJ site.

Age group	Highest TECR-Contributing Source	Highest HI-Contributing Source	Consistency with Adult Ranking
Infant	Metal processing products, chemicals and chemical products manufacturing	Metal processing products, chemicals and chemical products manufacturing	Yes
Young children	Metal processing products, chemicals and chemical products manufacturing	Metal processing products, chemicals and chemical products manufacturing	Yes
Elementary school children	Metal processing products, chemicals and chemical products manufacturing	Metal processing products, chemicals and chemical products manufacturing	Yes
Adolescent	Metal processing products, chemicals and chemical products manufacturing	Metal processing products, chemicals and chemical products manufacturing	Yes
Adults	Metal processing products, chemicals and chemical products manufacturing	Metal processing products, chemicals and chemical products manufacturing	Reference
Elderly	Metal processing products, chemicals and chemical products manufacturing	Metal processing products, chemicals and chemical products manufacturing	Yes

https://doi.org/10.3390/toxics14090799

Table S24. Age-specific source-oriented health risk rankings at the BHS site.

Age Group	Highest TECR-Contributing Source	Highest HI-Contributing Source	Consistency with Adult Ranking
Infant	Chemicals and chemical products manufacturing	Background hydrocarbons	Yes
Young children	Chemicals and chemical products manufacturing	Background hydrocarbons	Yes
Elementary school children	Chemicals and chemical products manufacturing	Background hydrocarbons	Yes
Adolescent	Chemicals and chemical products manufacturing	Background hydrocarbons	Yes
Adults	Chemicals and chemical products manufacturing	Background hydrocarbons	Reference
Elderly	Chemicals and chemical products manufacturing	Background hydrocarbons	Yes

Table S25. Age-specific source-oriented health risk rankings at the JN site.

Age Group	Highest TECR-Contributing Source	Highest HI-Contributing Source	Consistency with Adult Ranking
Infant	Vehicle exhuast: Acetylene-rich	Textile product manufacturing; including clothing	Yes
Young children	Vehicle exhuast: Acetylene-rich	Textile product manufacturing; including clothing	Yes
Elementary school children	Vehicle exhuast: Acetylene-rich	Textile product manufacturing; including clothing	Yes
Adolescent	Vehicle exhuast: Acetylene-rich	Textile product manufacturing; including clothing	Yes
Adults	Vehicle exhuast: Acetylene-rich	Textile product manufacturing; including clothing	Reference
Elderly	Vehicle exhuast: Acetylene-rich	Textile product manufacturing; including clothing	Yes

Table S26. Descriptive statistics of the concentrations of the 16 selected VOCs at each measurement site.

VOCs	Measurement Sites			
	GS	GJ	BHS	JN
Ethane	5.09 (0.50-79.05)	4.09 (0.20-189.85)	2.34 (0.05-35.70)	6.86 (0.35-130.85)
Ethylene	1.97 (0.05-30.70)	1.30 (0.05-46.00)	0.49 (0.05-10.00)	2.16 (0.05-30.15)
Propane	7.25 (0.03-1435.23)	2.77 (0.13-88.93)	1.85 (0.03-31.13)	5.74 (0.07-336.37)
Propylene	0.77 (0.03-354.20)	0.51 (0.03-10.23)	0.39 (0.03-11.67)	0.71 (0.03-10.83)
Isobutane	2.89 (0.03-314.00)	0.91 (0.03-39.40)	0.57 (0.03-21.00)	2.16 (0.05-238.80)
n-Butane	7.67 (0.03-826.40)	1.76 (0.05-137.93)	0.94 (0.03-26.65)	3.81 (0.05-380.75)
Acetylene	1.18 (0.05-30.20)	0.56 (0.05-77.85)	0.21 (0.05-9.45)	0.95 (0.05-15.70)
Isopentane	1.13 (0.02-119.00)	0.83 (0.02-267.38)	0.47 (0.02-19.28)	1.53 (0.04-20.00)
n-Pentane	0.72 (0.02-35.42)	0.57 (0.02-257.04)	0.34 (0.02-29.74)	1.06 (0.02-59.88)
Hexane	0.52 (0.02-20.68)	0.35 (0.02-13.75)	0.33 (0.02-18.33)	0.70 (0.02-23.75)
Benzene	0.35 (0.02-42.05)	0.25 (0.02-10.77)	0.29 (0.02-15.97)	0.40 (0.02-3.70)
Toluene	4.41 (0.11-357.57)	2.00 (0.03-34.96)	1.22 (0.03-29.93)	3.68 (0.14-79.33)
Ethylbenzene	1.08 (0.04-202.50)	0.43 (0.01-32.08)	0.20 (0.01-68.15)	0.65 (0.01-52.58)
m,p-Xylene	1.46 (0.03-118.26)	0.54 (0.01-54.34)	0.21 (0.01-27.83)	0.80 (0.01-30.58)
o-Xylene	0.56 (0.04-64.49)	0.21 (0.01-8.24)	0.07 (0.01-5.39)	0.29 (0.01-10.84)
n-Dodecane	0.24 (0.02-69.54)	0.07 (0.01-8.83)	0.04 (0.01-3.30)	0.11 (0.01-5.46)

Values are presented as mean (minimum–maximum). Minimum concentrations represent the lowest detected concentrations, excluding non-detected values recorded as zero.

https://doi.org/10.3390/toxics14090799

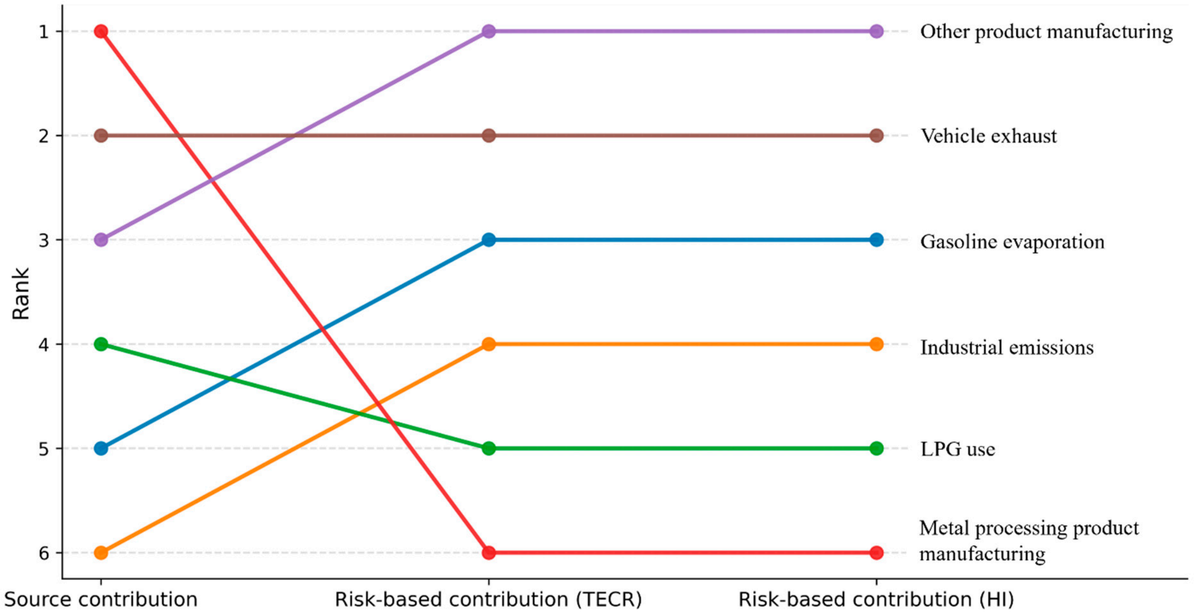

Figure S5. Comparison of source and risk-based contribution rankings of VOC sources at GS.

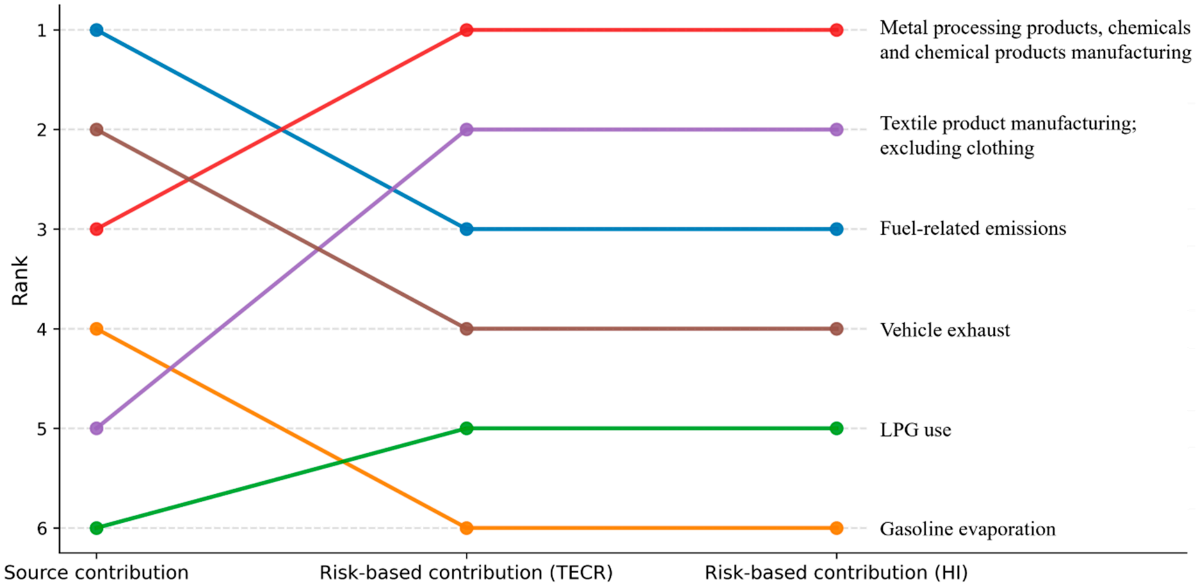

Figure S6. Comparison of source and risk-based contribution rankings of VOC sources at GJ.

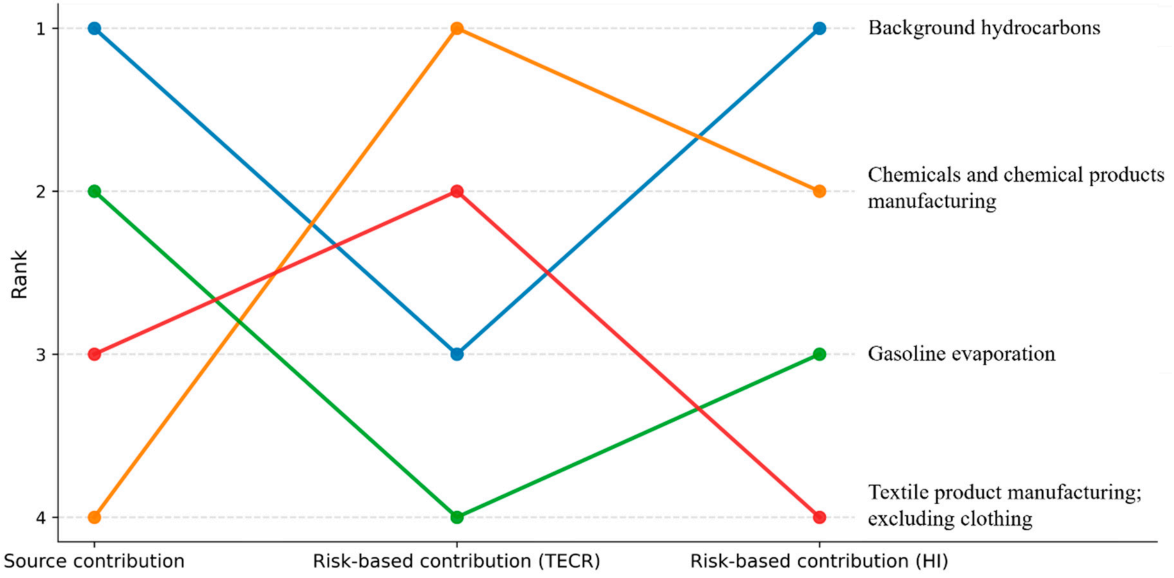

Figure S7. Comparison of source and risk-based contribution rankings of VOC sources at BHS.

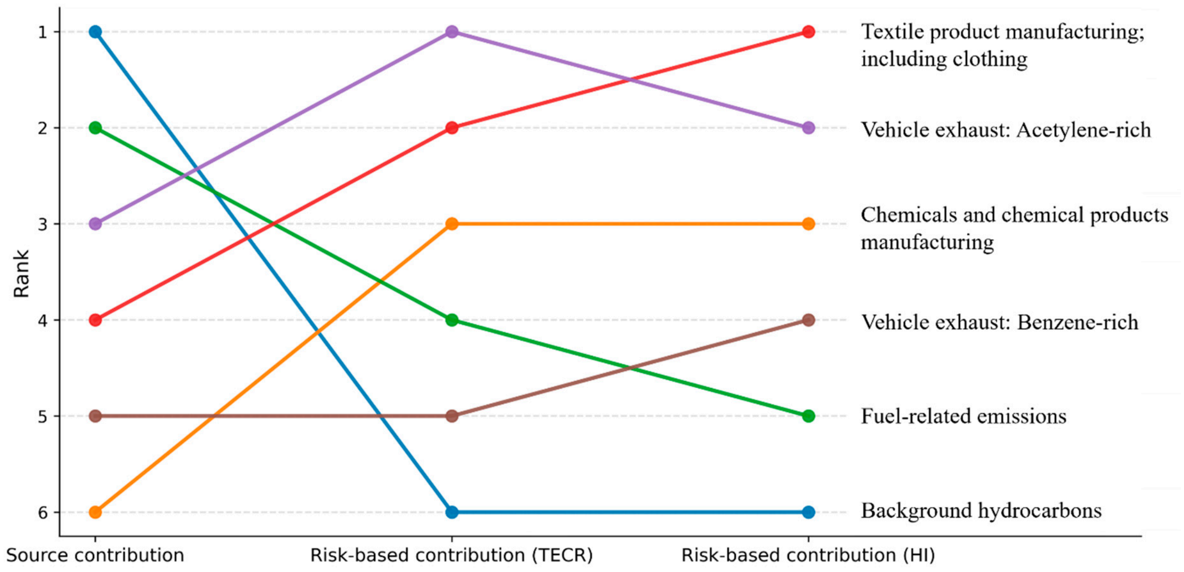

Figure S8. Comparison of source and risk-based contribution rankings of VOC sources at JN.

Disclaimer/Publisher's Note: The statements, opinions and data contained in all publications are solely those of the individual author(s) and contributor(s) and not of MDPI and/or the editor(s). MDPI and/or the editor(s) disclaim responsibility for any injury to people or property resulting from any ideas, methods, instructions or products referred to in the content.
